# ZFP36L2 suppresses mTORc1 through a P53-dependent pathway to prevent peripartum cardiomyopathy in mice

**DOI:** 10.1172/JCI154491

**Published:** 2022-05-16

**Authors:** Hidemichi Kouzu, Yuki Tatekoshi, Hsiang-Chun Chang, Jason S. Shapiro, Warren A. McGee, Adam De Jesus, Issam Ben-Sahra, Zoltan Arany, Jonathan Leor, Chunlei Chen, Perry J. Blackshear, Hossein Ardehali

**Affiliations:** 1 Department of Medicine, Northwestern University Feinberg School of Medicine, Chicago, Illinois, USA.; 2 Feinberg Cardiovascular and Renal Research Institute and; 3 Department of Biochemistry, Northwestern University, Chicago, Illinois, USA.; 4 Department of Medicine, University of Pennsylvania, Philadelphia, Pennsylvania, USA.; 5 Cardiovascular Research Institute, Tel Aviv University and Sheba Medical Center, Tel Aviv, Israel.; 6 Signal Transduction Laboratory, National Institute of Environmental Health Sciences, Research Triangle Park, North Carolina, USA.

**Keywords:** Cardiology, Cell Biology, Cardiovascular disease, Heart failure, p53

## Abstract

Pregnancy is associated with substantial physiological changes of the heart, and disruptions in these processes can lead to peripartum cardiomyopathy (PPCM). The molecular processes that cause physiological and pathological changes in the heart during pregnancy are not well characterized. Here, we show that mTORc1 was activated in pregnancy to facilitate cardiac enlargement that was reversed after delivery in mice. mTORc1 activation in pregnancy was negatively regulated by the mRNA-destabilizing protein ZFP36L2 through its degradation of *Mdm2* mRNA and P53 stabilization, leading to increased SESN2 and REDD1 expression. This pathway impeded uncontrolled cardiomyocyte hypertrophy during pregnancy, and mice with cardiac-specific *Zfp36l2* deletion developed rapid cardiac dysfunction after delivery, while prenatal treatment of these mice with rapamycin improved postpartum cardiac function. Collectively, these data provide what we believe to be a novel pathway for the regulation of mTORc1 through mRNA stabilization of a P53 ubiquitin ligase. This pathway was critical for normal cardiac growth during pregnancy, and its reduction led to PPCM-like adverse remodeling in mice.

## Graphical abstract

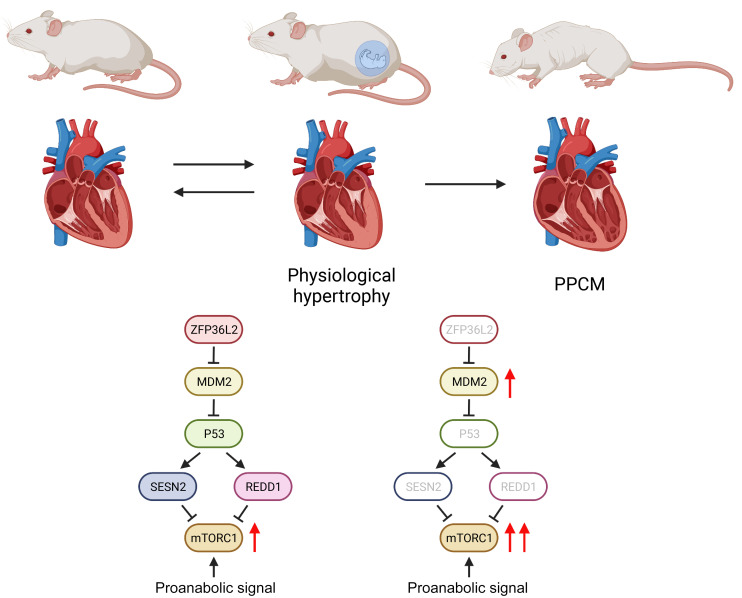

## Introduction

Pregnancy is associated with a number of changes to cardiovascular function, including increases in cardiac output, arterial compliance, and extracellular fluid volume (1). In addition, left ventricular (LV) wall thickness and mass increase by 28% and 52%, respectively (2). These adjustments are made to meet the increased metabolic demands of both the mother and the fetus as well as to ensure adequate circulation for fetal growth and development (1). However, alterations to normal cardiovascular physiology in pregnancy can lead to pregnancy-associated heart disorders, including peripartum cardiomyopathy (PPCM). PPCM is a form of cardiomyopathy that occurs in the last trimester of pregnancy and up to 6 months postpartum; it is defined by a decrease of the LV ejection fraction (EF) and congestive heart failure (3). If untreated, PPCM can lead to death. The etiology of PPCM is unknown; however, several mechanisms, including autoimmunity, systemic viral illness, micronutrient deficiency, and genetics, have been proposed (4, 5). Recent studies have indicated that defects in cardiac angiogenic balance also contribute to PPCM (6, 7). However, a lack of robust rodent models has limited mechanistic insight into the etiology of this disease.

Zinc finger protein (ZFP) 36 like 2 (ZFP36L2, also known as TIS11D, ERF2, and BRF2) is a member of the ZFP family of mRNA binding proteins, which also includes tristetraprolin (also known as ZFP36) and ZFP36L1. Members of this family bind to specific AU-rich elements (AREs) in the 3′ untranslated region (UTR) of mRNA molecules, causing their degradation (8). Global deletion of *Zfp36l2* in mice does not lead to embryonic lethality (unlike deletion of *Zfp36l1*); however, newborn mice die within 2 weeks after birth and suffer from pancytopenia as a result of a deficiency in hematopoietic stem cells in the liver (9). Mutant mice expressing an N-terminal truncated form of ZFP36L2 exhibit early developmental defects and growth arrest at the 2-cell embryonic stage (10). Simultaneous deletion of *Zfp36l1* and *Zfp36l2* in thymus during early period of its development led to the development of Notch1-dependent T cell acute lymphoblastic leukemia (11). Furthermore, tissue-specific deletion of *Zfp36l1* and *Zfp36l2* in pro–B cells demonstrated that these two proteins are critical for quiescence after cellular expansion by precursor B cell receptor (12). Thus, ZFP36L2 appears to be critical in the development of certain cells in the body. However, its function in the heart and the mechanism by which it regulates cardiomyocyte physiology is not entirely understood.

The mechanistic target of rapamycin (mTOR) is a master regulator of protein synthesis, glucose metabolism, and autophagy (13). The role of mTOR in various murine models of heart disease has been studied extensively. A number of studies have looked at mTOR complex 1 (mTORc1) hyperactivation in the heart. Cardiac-specific deletion of *Tsc1* or *Tsc2* leads to death from heart failure at 6 months and 10 months of age, respectively (14, 15), while *Sesn1/2/3* triple-KO mice died during the neonatal period owing to failure to activate autophagy (16). These studies all indicate that mTORc1 hyperactivation is detrimental to normal embryonic development and cardiac function. For models of mTORc1 suppression, both genetic and pharmacological approaches have been employed. Cardiac-specific deletion of *Mtor* causes lethality before the end of gestation (17) or within 3 weeks after birth (18), while deletion of the gene in the heart after birth causes lethal cardiomyopathy 5 weeks after induction (19). Treatment with rapamycin has been shown to extend life span in multiple organisms (20). Additionally, it regressed cardiac hypertrophy induced by trans-aortic constriction (TAC) and age-related cardiac hypertrophy (21, 22). Collectively, these studies demonstrate that the heart requires precise control of mTORc1 activity within a homeostatic range, and deletion of either the central mTORc1 components or its repressors leads to substantial mTORc1 dysregulation and severe damage to the heart.

In this paper, we studied the effects of cardiac-specific deletion of *Zfp36l2* on cardiac function. To our surprise, mice with deletion of this gene did not have a marked phenotype, except for in female mice after pregnancy, which displayed higher mortality and cardiac dysfunction. To define the mechanism for this phenotype, we undertook unbiased transcriptomic analyses, which showed alterations in the expression of key genes in the mTORc1 pathway with *Zfp36l2* deletion. Further studies narrowed this mechanism to a reduction in P53-mediated SESN2 and REDD1 expression, leading to chronic mTORc1 activation. Here, we showed that ZFP36L2 bound to and degraded the mRNA of the E3 protein-ubiquitin ligase MDM2; thus, *Zfp36l2* deletion leads to upregulation of MDM2 and P53 destabilization. We also showed that reversing mTORc1 hyperactivation in female mice with cardiac-specific *Zfp36l2* KO improved cardiac function. Overall, our results indicate that mRNA decay plays a major role in the regulation of the P53/mTORc1 pathway and that loss of ZFP36L2 in the heart leads to hyperactivation of mTORc1 and a PPCM-like phenotype in mice.

## Results

### Zfp36l2 deletion in the heart leads to a phenotype consistent with PPCM.

**Figure 1:**
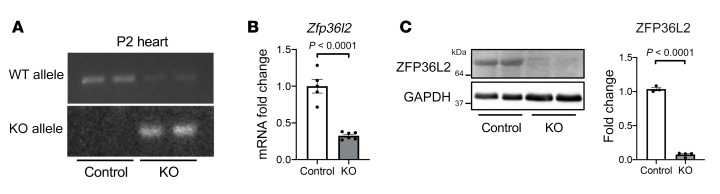
Successful cardiac *Zfp36l2* deletion in mice. (**A**) DNA (*n* = 2), (**B**) mRNA (*n* = 5–6), and (**C**) protein levels (*n* = 3–4) of *Zfp36l2* (**A**) at postnatal day 2 in the whole heart or (**B** and **C**) at 8 weeks old in isolated cardiomyocytes of cardiac-specific *Zfp36l2*-KO and control mice, confirming deletion of the gene in these animals. Data were analyzed by unpaired Student’s *t* test.

Previous studies have demonstrated that 2 members of the ZFP36 family play a role in cardiac function; deletion of *Zfp36l1* and *Zfp36* (also known as *Ttp*) causes embryonic heart defects (23, 24) and cardiomyopathy in iron deficiency (25), respectively. While these proteins share structural homology (26), it is not clear whether deletion of *Zfp36l2* would have any effects on cardiac function and what major mRNA molecules ZFP36L2 targets. Therefore, we generated cardiac-specific deletion of *Zfp36l2* by crossing mice with LoxP sequences flanking exon 2 of *Zfp36l2* (27) with αMHC-Cre mice. The control mice for our subsequent experiments were *Zfp36l2*^fl/fl^ Cre^–^. Cre expression resulted in deletion of *Zfp36l2* at birth, which was confirmed at the genomic, RNA, and protein level (Figure 1). As expected, hearts from *Zfp36l2*-KO mice did not display any change in the levels of ZFP36 or ZFP36L1 proteins (Supplemental Figure 1, A and B; supplemental material available online with this article; https://doi.org/10.1172/JCI154491DS1). Examination of the hearts showed no gross and histological abnormalities, and although the cross-sectional area (CSA) was slightly increased in the KO mice, the degree of fibrosis was similar between the groups (Supplemental Figure 1, C–E). Additionally, *Zfp36l2*-KO male and female mice did not display a reduction in EF at 10, 17, and 23 weeks of age (Supplemental Figure 2), supporting that the deletion of this gene does not alter cardiac function at baseline.

**Figure 2:**
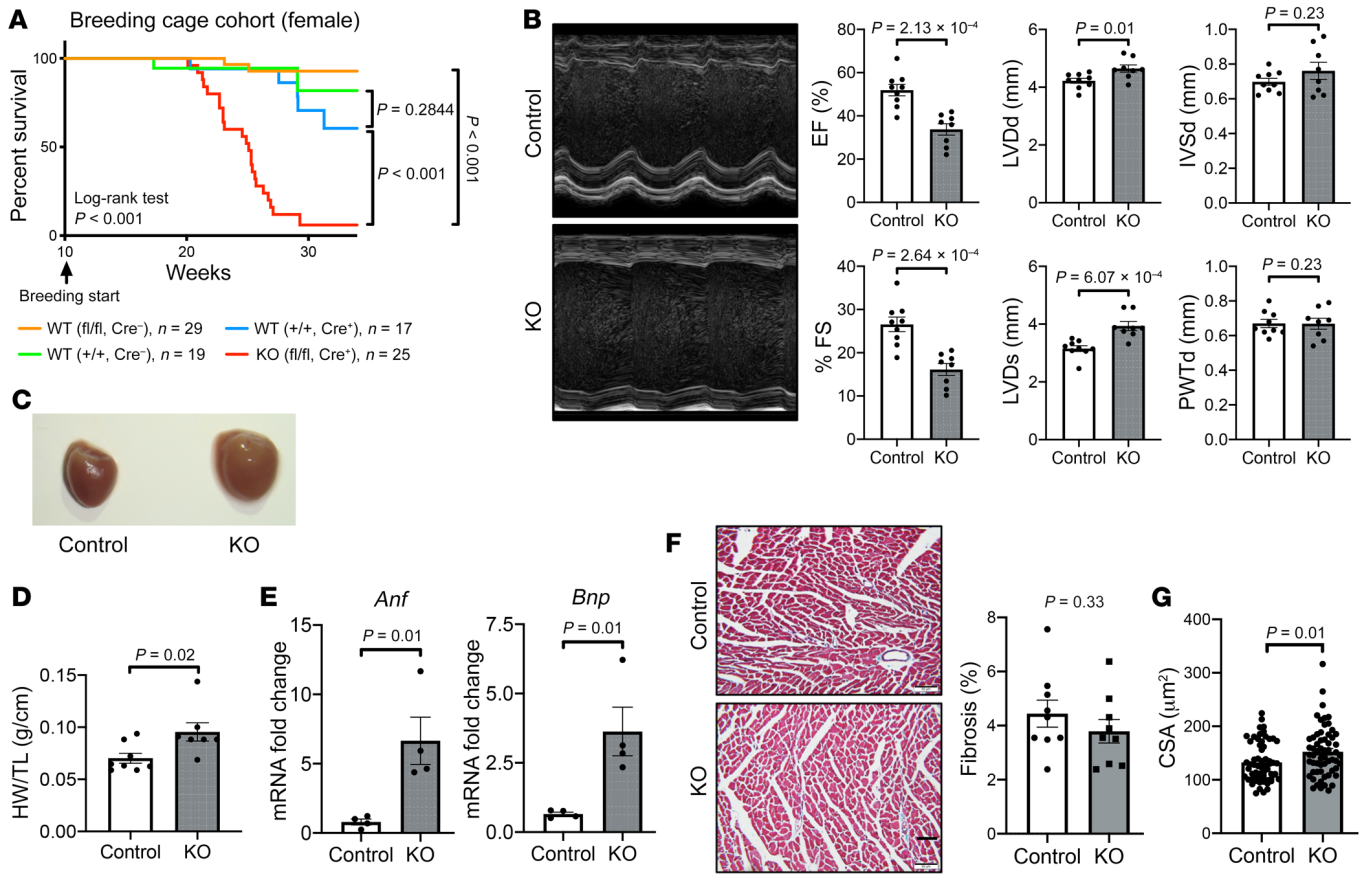
Cardiac *Zfp36l2* deletion induces heart failure. (**A**) Survival of *Zfp36l2*-KO mice and control mice after an average of 2 pregnancies and their deliveries. (**B**) Echo measurements in *Zfp36l2*-KO and control mice 7 days after first delivery (*n* = 8–9). EF, ejection fraction; FS, fractional shortening; LVDd, LV diastolic diameter; LVDs, LV systolic diameter. (**C**) Gross cardiac examination, (**D**) heart weight divided by tibial length (HW/TL) (*n* = 7–8), and (**E**) *Anf* and *Bnp* levels (*n* = 4) in *Zfp36l2*-KO and control mice 7 days after the first pregnancy. (**F**) Histology and assessment of the degree of fibrosis in *Zfp36l2*-KO and control littermates 7 days after first delivery (*n* = 3 mice and 3 sections per mouse). Scale bar: 50 μm. (**G**) Cross-sectional area (CSA) in *Zfp36l2*-KO and control littermates 7 days after first delivery (*n* = 3 mice and 20 cardiomyocytes per mouse). Data were analyzed by log-rank test with Bonferroni’s post hoc analyses for multiple group comparison (**A**) or unpaired Student’s *t* test (**B**, **D**–**F**, and **G**).

Pregnant female *Zfp36l2*-KO mice displayed a high mortality after delivery, and more than 90% of the mice died by 30 weeks of age (Figure 2A). The increased mortality in pregnant female *Zfp36l2*-KO mice was due to cardiac dysfunction, as the KO mice displayed markedly reduced cardiac function 7 days after delivery from first pregnancy (Supplemental Figure 3). Cardiac-specific deletion of *Zfp36l2* had no effect on the fertility of the mice, as both control and KO dams had a comparable number of deliveries and time to first delivery (Supplemental Figure 4, A and B). The KO mice also showed reduced EF and fractional shortening and increased LV diastolic and systolic diameters (LVDd and LVDs, respectively) 7 days after the first pregnancy (Figure 2B). Examination of the hearts after the first pregnancy revealed enlarged hearts (Figure 2C) and an increased heart weight/tibial length ratio (Figure 2D). Additionally, mRNA of markers of heart failure, *Anf* and *Bnp*, were increased in the hearts of *Zfp36l2-*KO mice after pregnancy (Figure 2E); however, there was no significant change in overall cardiac histologic structure or degree of fibrosis (Figure 2F). *Zfp36l2*-KO hearts also displayed higher CSA, consistent with possible cardiomyocyte hypertrophy (Figure 2G). The effects of second pregnancy were even more pronounced, as mice with *Zfp36l2* deletion displayed a more pronounced reduction in EF and fractional shortening and an increase in LVDd and LVDs (Supplemental Figure 5A), in addition to larger hearts (Supplemental Figure 5B). The rapid decline in cardiac function after pregnancy and the lack of tissue fibrosis indicate that deletion of *Zfp36l2* in mice leads to a phenotype consistent with PPCM, which is progressive and causes increased mortality. Although, all ZFP36 family members recognize the same ARE sequences in the 3′-UTR of transcripts and can share targets, cardiac-specific deletion of *Zfp36* and *Zfp36l1* did not result in mortality after pregnancy, demonstrating that the PPCM phenotype is due to a specific function of ZFP36L2 (Supplemental Figure 6).

### Deletion of Zfp36l2 in the heart leads to the activation of the mTORc1 pathway.

**Figure 3:**
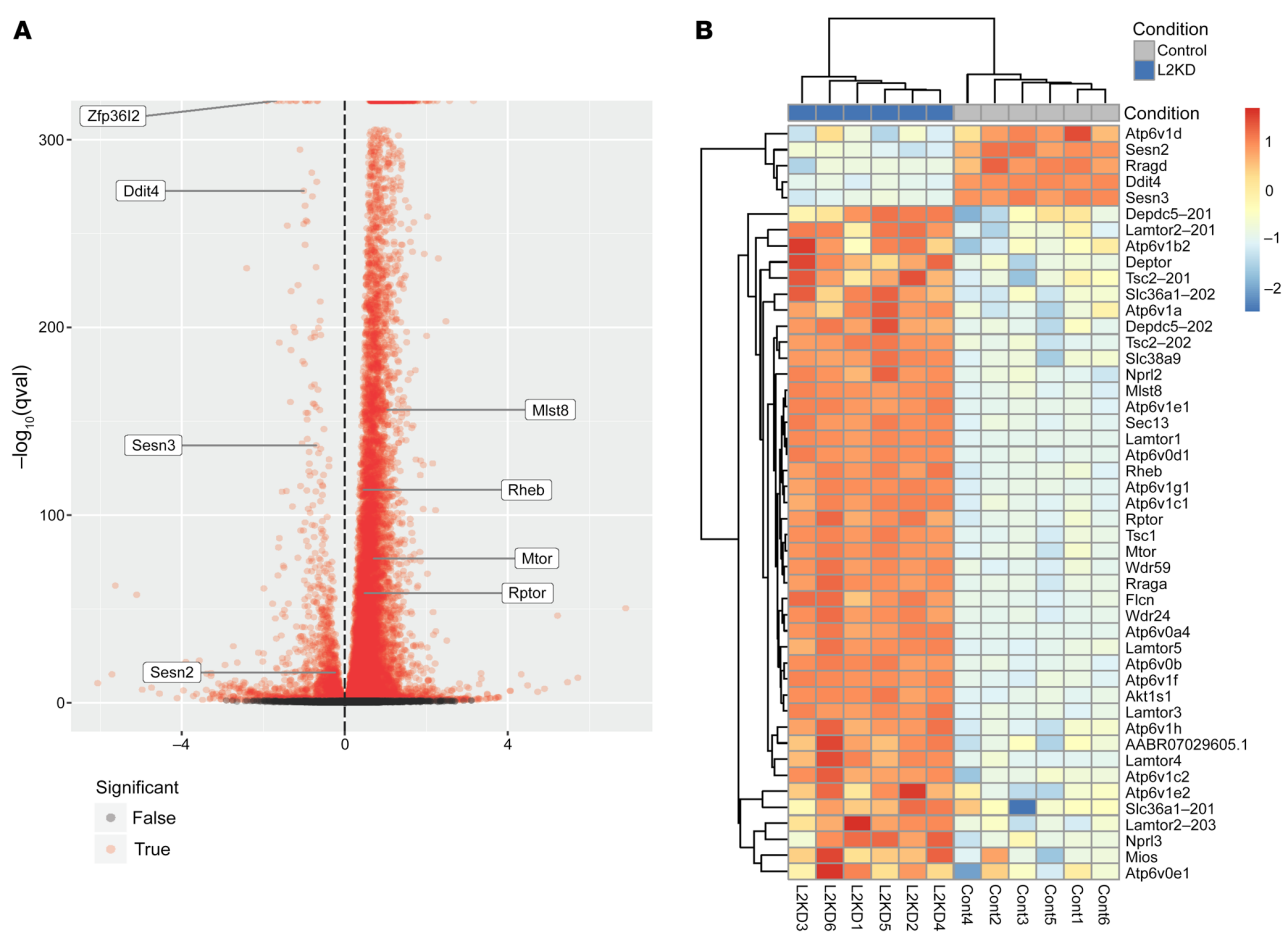
RNA-Seq of H9c2 cells treated with *Zfp36l2* or control siRNA. (**A**) Volcano plot of transcripts from the *Zfp36l2* KD versus control RNA-Seq experiment. Transcripts were normalized to *Actb* and *Hprt1*, and depicted are the –log_10_ transformation of the adjusted *P* values versus the “beta value” estimated by sleuth-ALR. Highlighted genes include *Zfp36l2* to confirm the KD, and other genes in the mTOR signaling pathway that had 1 major transcript significantly upregulated or downregulated. (**B**) Heatmap plot of differentially expressed transcripts from the mTOR signaling pathway estimated with *Zfp36l2* KD (*n* = 6).

We next studied the mechanism by which *Zfp36l2* deletion causes pathological effects in the heart. We performed RNA-Seq in H9c2 cardiomyoblasts treated with control or *Zfp36l2* siRNA. The in vitro approach for this analysis was chosen over using intact hearts from the KO mice to avoid both the unwanted side effects of cardiomyopathy and contamination from noncardiac cells on gene expression. The efficiency of knockdown (KD) was confirmed at the mRNA and protein level (Supplemental Figure 7). It is predicted that ZFP36L2 binds to and causes degradation of a large number of mRNAs (27, 28); thus, we expected that the unbiased RNA-Seq analysis would yield changes in a large number of mRNAs. Indeed, RNA-Seq of the cells with *Zfp36l2* KD showed an increase in 9765 mRNAs and a decrease in 1435 mRNAs compared with control cells (Figure 3A and Supplemental Table 1). We then performed pathway analysis of the transcripts that are significantly altered in the RNA-Seq data. This analysis revealed metabolic pathways, specifically genes involved in PI3K/mTORc1 signaling, as most significantly changed with *Zfp36l2* KD (Table 1 and Figure 3B). Additionally, analysis of the volcano plot revealed several positive regulators of the mTORc1 pathway to be upregulated, while a number of negative regulators to be downregulated (Figure 3). Thus, we further analyzed the RNA-Seq data for changes in mTORc1-related genes with *Zfp36l2* KD. Most of the genes demonstrated a significant increase in expression with downregulation of *Zfp36l2*, with the exception of 5 genes, including *Redd1* (also known as *Ddit4*), *Sesn2* and *Sesn3* (Figure 3B), which are negative regulators of mTORc1 and were decreased with *Zfp36l2* downregulation. Based on these results, we focused on the mTORc1 pathway for the mechanistic studies.

**Figure 4:**
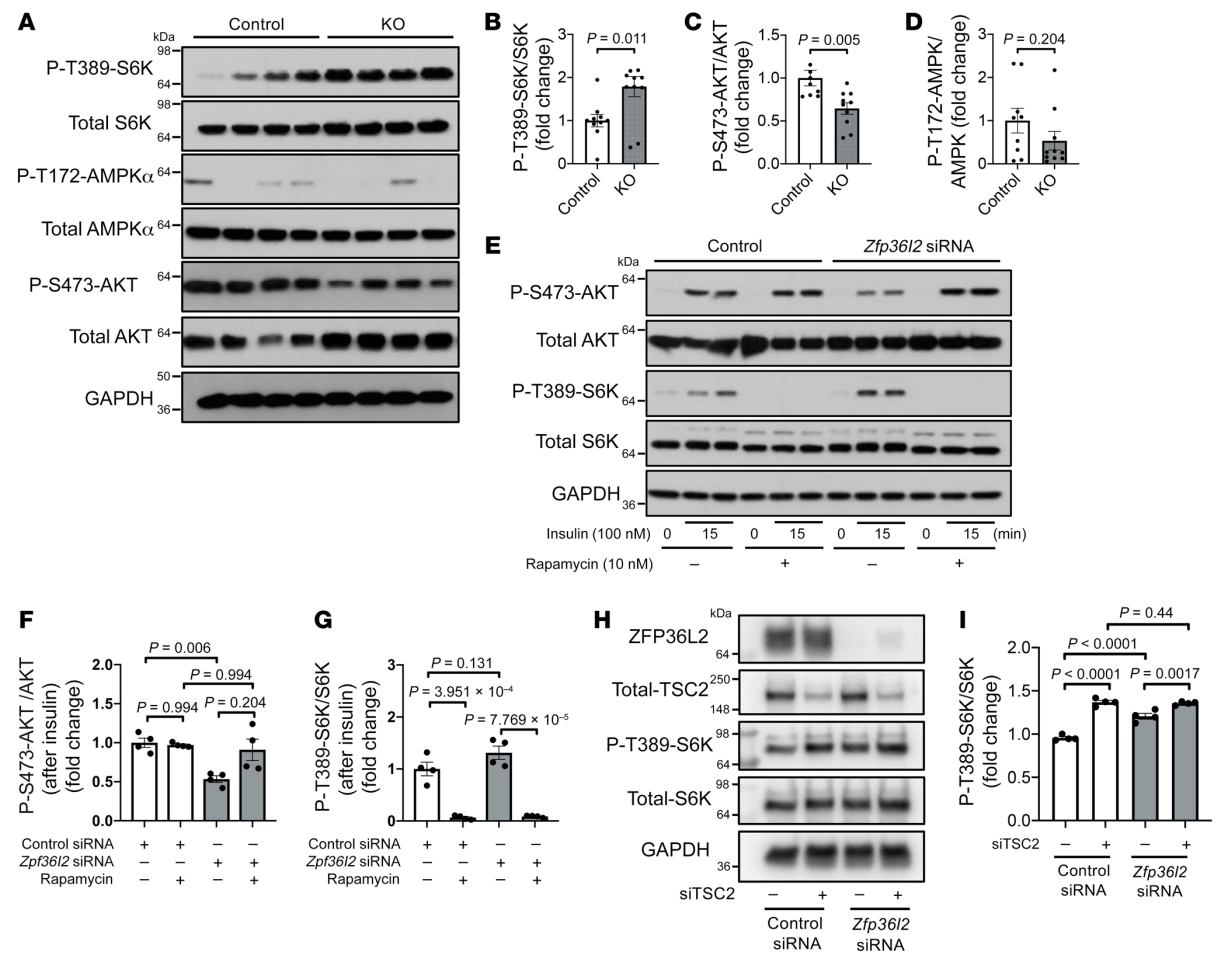
*Zfp36l2* deletion in the heart causes activation of the mTORc1 pathway. (**A**) Representative Western blot of heart extracts from control and *Zfp36l2*-KO mice, demonstrating increased mTOR activity with *Zfp36l2* deletion. (**B**–**D**) Summary of densitometry of Western blot analyses in **A**, (**B**) demonstrating increased p-P70S6K^T389^ (*n* = 10–11), (**C**) decreased p-AKT^S473^ (*n* = 9–10), and (**D**) no change in p-AMPK^T172^ (*n* = 9–10). (**E**) Representative Western blot of H9c2 cells treated with control or *Zfp36l2* siRNA in the presence and absence of insulin and 10 nM rapamycin. (**F** and **G**) Summary of densitometry of Western blot analysis in **E** (*n* = 4). (**H**) Representative Western blot of H9c2 cells treated with control or *Tsc2* siRNA with or without concurrent *Zfp36l2* KD. (**I**) Summary of densitometry of Western blot for p-P70S6K^T389^ in **H** (*n* = 4). Data were analyzed by unpaired Student’s *t* test (**B**–**D**) or 2-way ANOVA with Tukey’s test for multiple group comparison (**F**, **G**, and **I**).

To validate that mTORc1 activity is altered upon *Zfp36l2* KO, we measured the phosphorylation of P70S6K. *Zfp36l2*-KO hearts at 8 weeks of age displayed significantly higher p-P70S6K^T389^ levels compared with control hearts (Figure 4, A and B), indicating higher mTORc1 activity. Consistent with hyperactivated mTORc1, *Zfp36l2* KD in H9c2 cells led to increased ^35^S methionine incorporation into proteins (Supplemental Figure 8).

Downregulation of *Zfp36l2* also resulted in reduced AKT^S473^ phosphorylation (Figure 4, A and C). AKT^S473^ is a direct target of mTORc2, which is subject to negative regulation by mTORc1. To determine if the decrease in p-AKT^S473^ was due to an independent decrease in mTORc2 activity or a result of feedback inhibition from hyperactivated mTORc1, we treated cells with rapamycin and measured AKT^S473^ phosphorylation in response to insulin. Rapamycin treatment rescued AKT^S473^ phosphorylation in *Zfp36l2*-KD cells (Figure 4, E and F), and it similarly suppressed P70S6K^T389^ phosphorylation (Figure 4, E and G), indicating that mTORc1 activity is regulated by ZFP36L2 and the decrease in p-AKT^S473^ is due to mTORc2 inhibition by mTORc1. These results collectively suggest that deletion of *Zfp36l2* increases mTORc1 activity.

### mTORc1 regulation by ZFP36L2 is through TSC1/2 and amino acid sensing.

**Figure 5:**
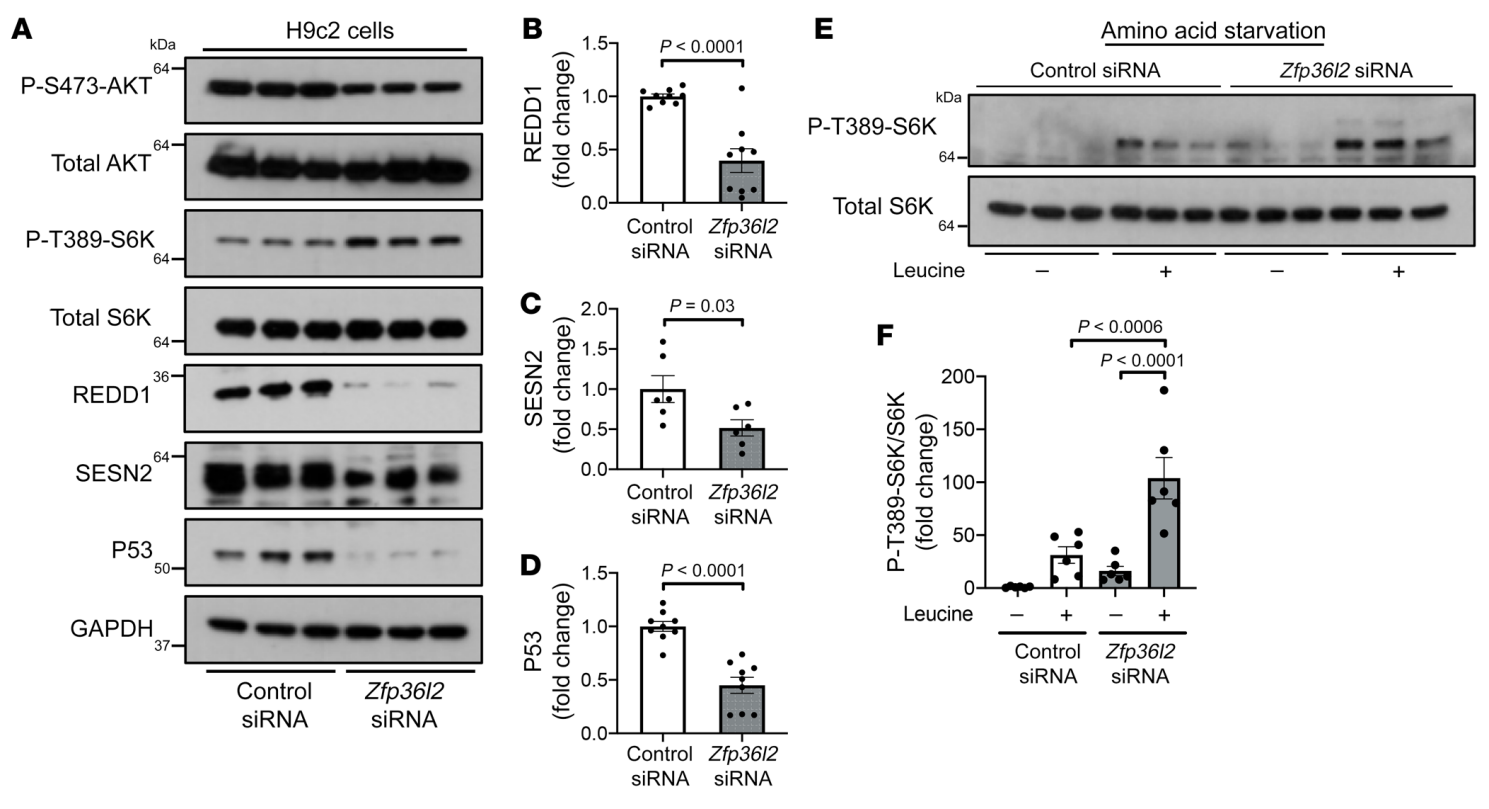
mTORc1 regulation by ZFP36L2 is through amino acid sensing and SESN2 and REDD1. (**A**) Representative Western blot of H9c2 cells treated with control or *Zfp36l2* siRNA and probed with p-AKT^S473^, p-P70S6K^T389^, SESN2, REDD1, and P53. (**B**–**D**) Summary of densitometry of Western blots in **A**, demonstrating decreased (**B**) REDD1 (*n* = 9), (**C**) SESN2 (*n* = 9), and (**D**) P53 (*n* = 9). (**E**) Representative Western blot and (**F**) summary of densitometry of p-P70S6K^T389^ (*n* = 6) in H9c2 cells with *Zfp36l2* KD under amino acid starvation and leucine supplementation. Data were analyzed by unpaired Student’s *t* test (**B**–**D**) or 2-way ANOVA with Tukey’s test for multiple group comparison (**F**).

The TSC1/2 complex is a major signaling hub for mTORc1 regulation by RHEB (29, 30). We first assessed whether the mechanism by which ZFP36L2 regulates mTORc1 activity requires a functional TSC complex by measuring P70S6K^T389^ phosphorylation in cells treated with *Tsc2* siRNA with and without concurrent *Zfp36l2* downregulation. *Tsc2* downregulation was sufficient to diminish the effects of *Zfp36l2* KD on mTORc1 activation (Figure 4, H and I), indicating that the regulation of mTORc1 by ZFP36L2 is through TSC2. Because mRNA levels of *Redd1* (a positive regulator of TSC2) were reduced in the RNA-Seq of *Zfp36l2-*KD cells (Figure 3B), we then measured protein levels of REDD1 with *Zfp36l2* KD and showed that it was reduced (Figure 5, A and B). Additionally, TSC2 localization to lysosomes, a process required for mTORc1 inhibition, was significantly decreased in *Zfp36l2*-KD cells (Supplemental Figure 9). These findings collectively indicate that ZFP36L2 regulates mTORc1 through REDD1, a negative regulator of TSC complex.

To rule out influence of ZFP36L2 on other TSC-mediated upstream regulators of mTORc1 activity, we measured the activity of the AKT and AMPK pathways after downregulation of *Zfp36l2*. Our results indicated similar increases in p-P70S6K^T389^ and p-AKT^S473^ upon insulin stimulation in control and *Zfp36l2*-KD cells (Supplemental Figure 10, A–C), indicating that mTORc1 regulation by ZFP36L2 is independent of growth factor signaling. We also studied whether changes in cellular energetics, signaled through AMPK, mediate mTORc1 regulation by ZFP36L2. p-AMPK^T172^ was not changed in the hearts of *Zfp36l2*-KO mice (Figure 4, A and D), suggesting that AMPK is also not required for the regulation of mTORc1.

mTORc1 activity is also modulated by the availability of amino acids (particularly leucine), which are sensed through the GATOR complexes and RAG GTPase (31, 32). Because regulation of mTORc1 by amino acids is independent of the TSC1/2 complex, we next determined whether ZFP36L2 also regulates mTORc1 through amino acid signaling. We first measured mTORc1 activity under total amino acid starvation and observed higher p-P70S6K^T389^ levels in *Zfp36l2*-KD cells (Figure 5, E and F). Additionally, leucine supplementation resulted in further hyperactivation of mTORc1 (Figure 5, E and F). Consistent with RNA-Seq data showing reduced *Sesn2* mRNA levels (Figure 3B), we observed reduced SESN2 protein levels in cells with ZFP36L2 downregulation (Figure 5, A and C). Additionally, ZFP36L2 is also regulated by amino acid starvation (Supplemental Figure 10D). Together, these findings indicate that loss of *Zfp36l2* regulates mTORc1 through amino acid sensing, in addition to the TSC1/2 pathway.

### ZFP36L2 inhibits mTORc1 through a P53-mediated pathway.

The results thus far indicate that ZFP36L2 inhibits mTORc1 independent of energy starvation (through AMPK) and growth factor signaling (through AKT) but involves the negative regulator of the TSC complex REDD1 and the leucine sensor SESN2. Because the levels of both REDD1 and SESN2 are reduced in cells with *Zfp36l2* KD, the regulation of these transcripts by ZFP36L2 is likely indirect. Previous studies implicated both SESN2 and REDD1 as downstream mediators of cellular stress response pathways (33, 34). To determine the protein(s) that mediate the ZFP36L2 signal to SESN2 and REDD1, we next studied the physiological consequences of this regulation and whether ZFP36L2 modulation alters cellular response to stress. Cells treated with *Zfp36l2* siRNA displayed increased cellular ROS and injury in response to H_2_O_2_ treatment (Supplemental Figure 11, A–C). Similar results were observed in cells treated with etoposide, which primarily induced DNA double-strand breaks (Supplemental Figure 11D). The increase in cell death with *Zfp36l2* KD was not due to higher levels of DNA damage, as the levels of phosphorylated histone H2AX, a marker of DNA strand breaks, were similar between control siRNA– and *Zfp36l2* siRNA–treated cells after doxorubicin (DOX) treatment (Supplemental Figure 12A).

The tumor suppressor protein P53, a major integrator of cellular stress signals, is induced following DNA damage as well as ROS insults, it is a key determinant of the balance between cellular repair versus cell death (35). Additionally, P53 is reported to regulate both REDD1 and SESN2 expression (36–38). Thus, we next examined whether P53 plays a role in ZFP36L2 regulation of SESN2 and REDD1. In *Zfp36l2*-KD cells, we observed reduced P53 protein levels at baseline (Figure 5, A and D), which persisted after etoposide, H_2_O_2_, or DOX treatment (Supplemental Figure 12, B–D).

**Figure 6:**
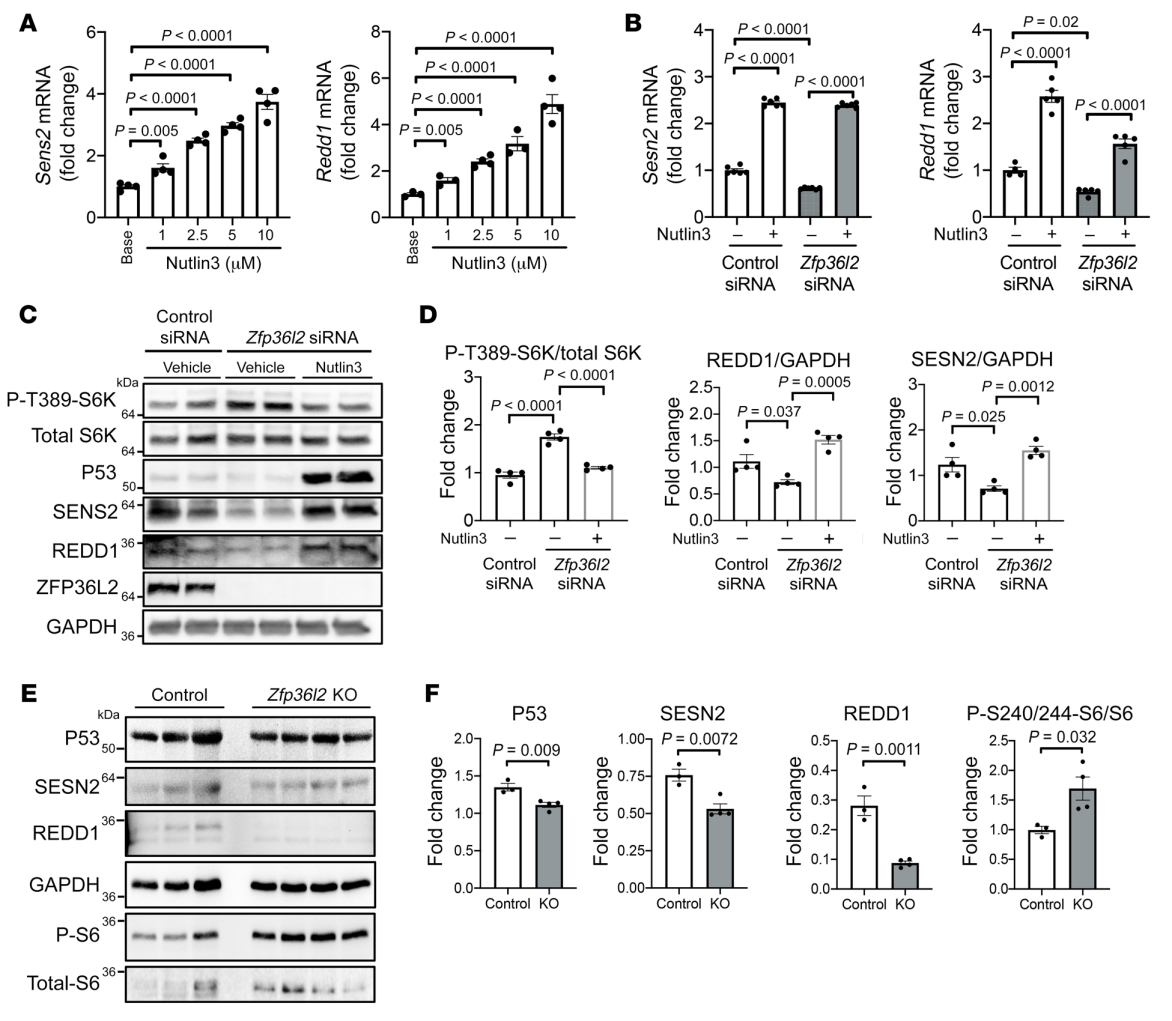
SESN2 and REDD1 regulation by ZFP36L2 is P53 dependent. (**A**) mRNA levels of *Sesn2* and *Redd1* after treating H9c2 cells with increasing concentrations of nutlin3, illustrating that nutlin3 increases both *Sesn2* and *Redd1* mRNA levels in a dose-dependent manner (*n* = 3–4). (**B**) *Sesn2* and *Redd1* mRNA levels in response to *Zfp36l2* KD and after treatment with nutlin3 (*n* = 4–6). (**C**) Representative Western blot of H9c2 cells treated with *Zfp36l2* siRNA in the presence and absence of nutlin3. (**D**) Summary of densitometry of Western blot analysis in **C** (*n* = 4), demonstrating that nutlin3 reverses the effects of *Zfp36l2* KD on the protein levels of P53, SESN2, and REDD1. (**E**) Western blot of P53, SESN2, REDD1, p-S6^S240/244^, and total S6 in cardiomyocytes isolated from mice with cardiac deletion of *Zfp36l2* and (**F**) summary of densitometry (*n* = 3–4). Data were analyzed by unpaired Student’s *t* test (**A** and **F**), 2-way ANOVA with Tukey’s test for multiple group comparison (**B**), or 1-way ANOVA with Tukey’s test for multiple group comparison (**D**).

We next determined whether changes in P53 levels are responsible for altered SESN2 and REDD1 expression. We measured the mRNA levels of these 2 proteins after treating H9c2 cells with increasing concentrations of nutlin3 (a chemical that inhibits the interaction between MDM2 and P53, preventing degradation of P53). Nutlin3 increased *Redd1* and *Sesn2* mRNA levels in a dose-dependent manner (Figure 6A). Additionally, nutlin3 reversed the reduction in *Redd1* and *Sesn2* mRNA in response to *Zfp36l2* KD (Figure 6B). Nutlin3 also reversed the reduction in REDD1 and SESN2 protein levels and the increase in P70S6K phosphorylation in response to *Zfp36l2* KD (Figure 6, C and D), indicating that P53 acts upstream of REDD1, SESN2, and, eventually, mTORc1. Furthermore, *P53*-KO mouse embryonic fibroblasts (MEFs) displayed no change in the mRNA and protein levels of REDD1 and SESN2 following KD of *Zfp36l2*, and KD of *Zfp36l2* in *P53* KO MEFs had no effect on mTORc1 activity (Supplemental Figure 13, A and B). Finally, we confirmed our findings by demonstrating that, in cardiomyocytes isolated from mice with cardiac deletion of *Zfp36l2*, protein levels of REDD1, SESN2, and P53 were decreased, while mTOR activity was increased (Figure 6, E and F), validating the RNA-Seq data in H9c2 cells (Figure 3). These results collectively indicate that P53 regulates ZFP36L2-mediated inhibition of mTORc1 through REDD1 and SESN2.

### ZFP36L2 binds to and degrades the mRNA for Mdm2.

**Figure 7:**
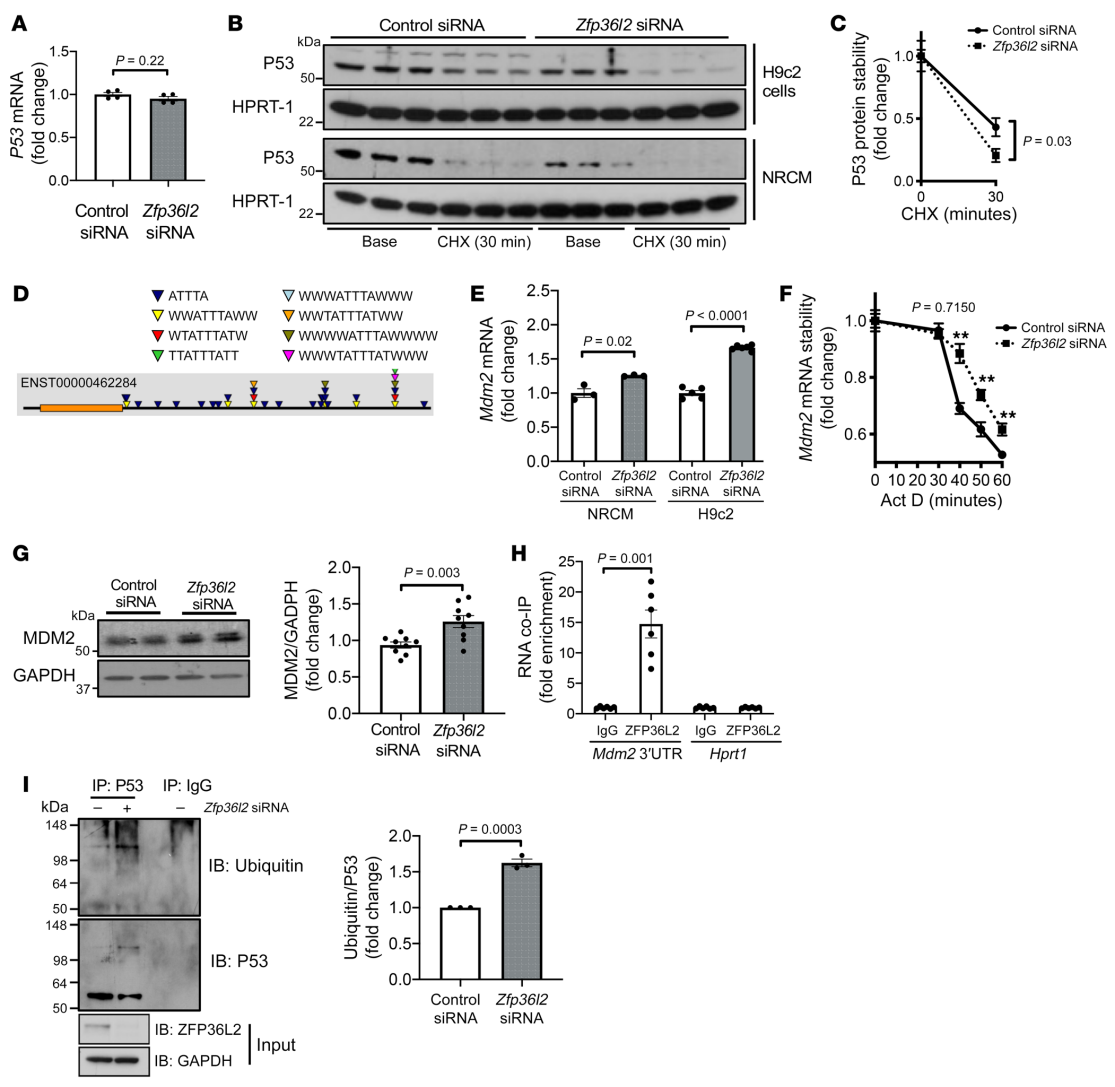
ZFP36L2 regulates P53 levels through its binding to and degradation of the *Mdm2* mRNA. (**A**) *P53* mRNA levels in H9c2 cells treated with control or *Zfp36l2* siRNA (*n* = 4). (**B**) Representative Western blot of P53 protein levels in H9c2 cells and neonatal rat cardiomyocytes (NRCMs) treated with control or *Zfp36l2* siRNA 30 minutes after cycloheximide (CHX) treatment (*n* = 3). (**C**) Time course of P53 protein stability in H9c2 cells treated with control or *Zfp36l2* siRNA after treatment with CHX (*n* = 7). (**D**) In silico analysis of the 3′-UTR of *Mdm2* mRNA demonstrated multiple consensus ARE sequences in its 3′-UTR. (**E**) Steady-state mRNA levels of *Mdm2* in H9c2 cells (*n* = 5–6) and NRCMs (*n* = 3) treated with control and *Zfp36l2* siRNA. (**F**) Time course of *Mdm2* mRNA stability in H9c2 cells treated with control or *Zfp36l2* siRNA and actinomycin D (*n* = 8). **P < 0.01. (**G**) MDM2 protein levels in H9c2 cells treated with control and *Zfp36l2* siRNA (*n* = 9). (**H**) RNA co-IP of *Mdm2* 3′-UTR with ZFP36L2 protein (*n* = 6). *Hprt1* was used as a negative control. (**I**) Assessment of P53 protein ubiquitination in H9c2 cells with control or *Zfp36l2* siRNA (*n* = 3). Data were analyzed by unpaired Student’s *t* test.

Although P53 protein levels were reduced (Figure 5, A and D), P53 mRNA levels did not change in response to *Zfp36l2* downregulation (Figure 7A). These findings are consistent with ZFP36L2 regulating P53 at the posttranslational level. Therefore, we measured protein stability of P53 by treating cells with cycloheximide to halt new protein synthesis. P53 protein decayed more rapidly in *Zfp36l2*-KD cells compared with that in control cells (Figure 7, B and C), indicating that P53 protein stability is reduced with *Zfp36l2* KD. This mechanism is not unique to proliferating cells, as terminally differentiated neonatal rat cardiomyocytes (NRCMs) demonstrated similar destabilization of P53 with *Zfp36l2* downregulation (Figure 7B). MDM2 is an E3 protein-ubiquitin ligase that leads to P53 degradation (39). We found that *Mdm2* mRNA has a number of consensus ARE sequences in its 3′-UTR, suggesting that ZFP36L2 may bind to its mRNA (Figure 7D). Steady-state *Mdm2* mRNA levels and stability were increased with *Zfp36l2* KD (Figure 7, E and F), and its protein levels were also increased with *Zfp36l2* KD (Figure 7G). Additionally, *Mdm2* 3′-UTR coimmunoprecipitated with ZFP36L2 protein (Figure 7H), indicating that ZFP36L2 binds to and degrades *Mdm2* mRNA. Finally, P53 protein ubiquitination is increased with *Zfp36l2* KD (Figure 7I), consistent with increased MDM2 protein levels. Thus, ZFP36L2 controls P53 protein levels by regulating the mRNA stability of E3 protein-ubiquitin ligase MDM2.

### mTORc1 is activated in murine pregnancy, and rapamycin treatment reverses the cardiomyopathy associated with Zfp36l2 deletion.

**Figure 8:**
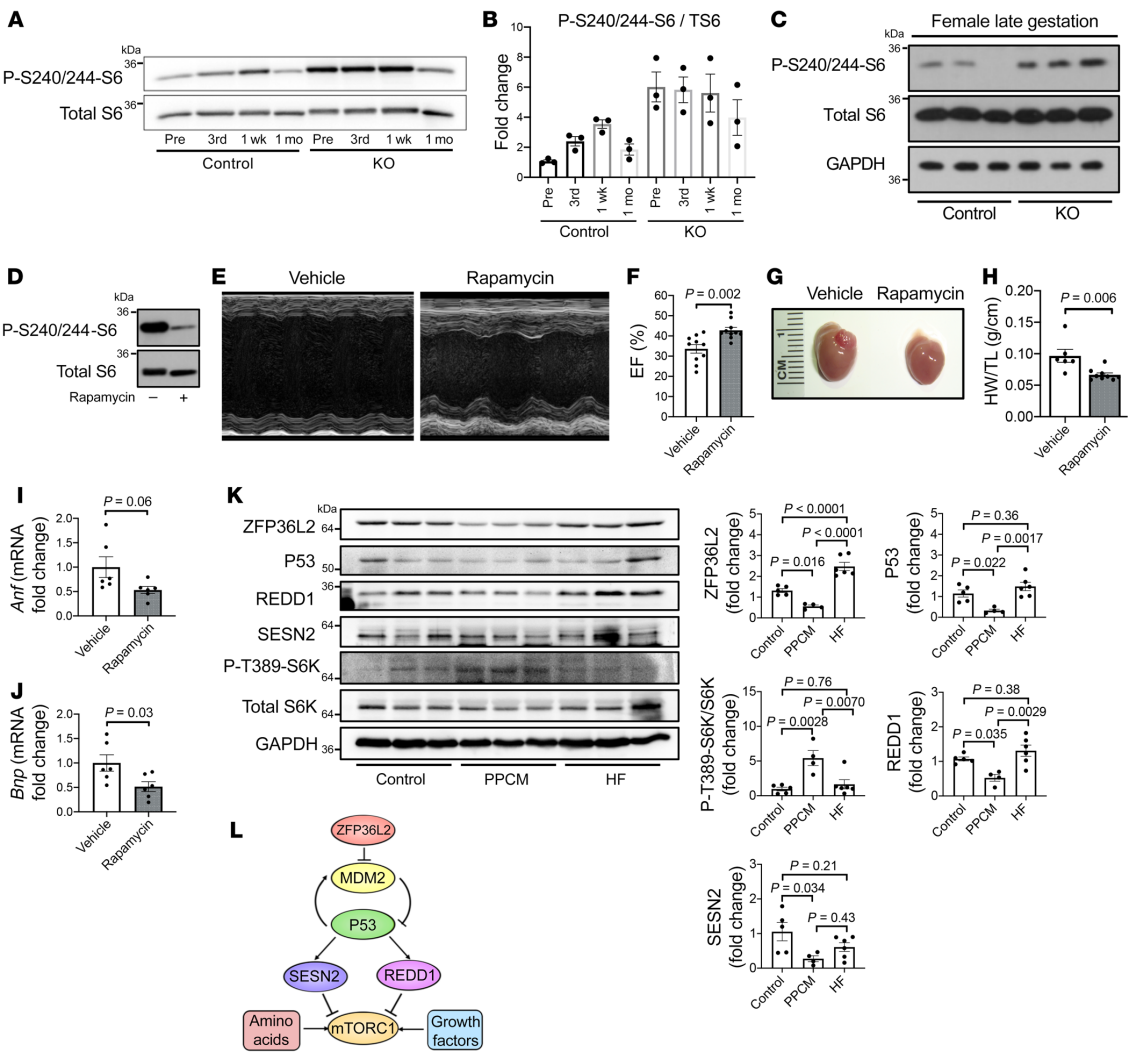
Rapamycin reverses the cardiac dysfunction that occurs in *Zfp36l2*-KO mice. (**A**) Representative Western blot of p-S6^S240/244^ and total S6 in WT and *Zfp36l2-*KO hearts in nonpregnant mice (Pre) as well as pregnant mice during the third trimester of the first pregnancy (3rd) and 1 week (1 wk) and 1 month (1 mo) after delivery. (**B**) Summary of densitometry of Western blot analysis in **A** (*n* = 3). (**C**) Western blot of p-S6^S240/244^ in the hearts of *Zfp36l2*-KO and control mice in the late gestation phase, E18–E19 (*n* = 3). (**D**) Effective reduction of mTOR activity in the hearts of mice treated with rapamycin, as assessed by measurement of p-S6^S240/244^. (**E**) 2D echo image and (**F**) summary bar graph of EF in *Zfp36l2*-KO mice after treatment with rapamycin or vehicle according to the protocol in Supplemental Figure 15A (*n* = 10). (**G**) Gross examination of the hearts of *Zfp36l2*-KO pregnant mice treated with a vehicle or rapamycin. (**H**) Heart weight to tibial length (HW/TL) ratio (*n* = 6–9) and (**I** and **J**) markers of heart failure (*Anf* and *Bnp*) (*n* = 6) in *Zfp36l2*-KO pregnant mice treated with a vehicle or rapamycin. (**K**) Protein levels of the components of the ZFP36L2/P53/REDD1/SESN2/mTOR pathway in patients with PPCM and other forms of heart failure (HF). (**L**) Graphic representation of the pathways through which ZFP36L2 regulates mTORc1 activity. Data were analyzed by unpaired Student’s *t* test (**F** and **H**–**J**) or 1-way ANOVA with Tukey’s test for multiple group comparison (**K**).

Given that our data demonstrated a role of ZFP36L2 in pregnancy-mediated cardiac pathology and that mTORc1 is regulated in this pathway, we next conducted 2 sets of in vivo experiments to determine (a) whether ZFP36L2 protein levels and mTORc1 activity are changed in pregnancy and (b) whether inhibition of mTOR can reverse the PPCM associated with *Zfp36l2* deletion. For the first set of experiments, we measured ZFP36L2 protein and mTOR activity in WT and *Zfp36l2*-KO mice in the third trimester of pregnancy and 1 and 4 weeks after pregnancy. Our results demonstrated that mTORc1 activity increased in the third trimester and continued to be elevated after 1 week of delivery, but it returned to normal 1 month after pregnancy (Figure 8, A and B). These results are consistent with those of previous reports that have shown mTOR activation during pregnancy (40). However, mTORc1 activity was substantially higher in *Zfp36l2*-KO mice during pregnancy and remained elevated after delivery (Figure 8, A and B). Cardiomyocytes from *Zfp36l2*-KO mice displayed larger CSA 1 week after pregnancy (Figure 2G). Additionally, ZFP36L2 levels did not appear to change during pregnancy (Supplemental Figure 14). These results indicate that baseline expression of ZFP36L2 is necessary to keep the mTORc1 activation in check during pregnancy in mice.

ZFP36L2 binds to a number of mRNA molecules and thus alters the activity of a number of pathways. To determine whether the postpartum cardiomyopathy associated with *Zfp36l2* deletion is due to its specific effects on the mTORc1 pathway and its hyperactivation, we treated female pregnant mice with 1 mg/kg subcutaneous rapamycin every other day in the third trimester of the first pregnancy (Supplemental Figure 15A). We initiated the treatment at the third trimester (E12.5) because inhibition of mTORc1 prior to the third trimester detrimentally affects fetal development (41). As expected, rapamycin injection did not alter fetal development, as indicated by a similar gain in body weight in mice receiving vehicle versus rapamycin (Supplemental Figure 15B). Hearts of pregnant female mice with *Zfp36l2* deletion sacrificed at E18–E19 of the first pregnancy displayed increased S6^S240/244^ phosphorylation (Figure 8C), while rapamycin treatment reduced mTORc1 activity (Figure 8D). Additionally, rapamycin significantly preserved LV systolic function in *Zfp36l2*-KO mice, as indicated by an increase in EF after the first pregnancy (Figure 8, E and F). The hearts of *Zfp36l2*-KO mice also displayed reduced LVDd and LVDs (Supplemental Figure 16, A–E). Finally, gross and histological examination of the hearts of pregnant mice with *Zfp36l2* deletion treated with rapamycin displayed reduced size, heart weight/tibial length, and markers of heart failure (*Anf* and *Bnp*) compared with mice given vehicle control (Figure 8, G–J). Rapamycin treatment also led to reduced cardiomyocyte CSA without altering cardiac fibrosis (Supplemental Figure 17). The effects of rapamycin were specific to pregnant *Zfp36l2*-KO mice, because nonpregnant *Zfp36l2*-KO mice and control pregnant mice did not show any change in their cardiac function after treatment with rapamycin (Supplemental Figure 18). These results indicate that increased mTORc1 activity can lead to the development of PPCM, and rapamycin treatment can be a potential therapy for this disease.

To determine whether changes in the components of the ZFP36L2/P53/REDD1/SESN2/mTOR pathway are specific to PPCM, we assessed the levels of these proteins in other forms of cardiomyopathy. Our results demonstrated that the components of this pathway were not altered in ischemic cardiomyopathy that occurred after myocardial infarction (Supplemental Figure 19) and pressure overload by TAC (Supplemental Figure 20). Finally, we subjected *Zfp36l2*-KO mice and their littermate controls to TAC and assessed their cardiac function. There was no difference in cardiac function after TAC between control and *Zfp36l2*-KO mice (Supplemental Figure 21). Collectively, these results indicate that the effects of *Zfp36l2* deletion on cardiac function are specific to PPCM and not other forms of cardiomyopathy.

We next sequenced the coding region of the *ZFP36L2* gene in patients with PPCM to identify any potential mutations that might contribute to this disease in humans. It is important to note that this approach does not identify mutations within introns or the regulatory portion of the *ZFP36L2* gene. Although we identified some variants in *ZFP36L2* in several patients (Supplemental Table 2), these variations are not rare or predicted to be pathological, as determined on the gnomAD website (https://gnomad.broadinstitute.org/). Thus, we concluded that mutations within the coding region of the *ZFP36L2* gene are unlikely to cause PPCM.

We next obtained cardiac tissue samples from control participants and patients with PPCM to assess whether there was a change in the levels of ZFP36L2 protein in PPCM. Characteristics of patients from whom these samples were obtained are included in Supplemental Table 3. PPCM cardiac tissues displayed lower ZFP36L2 protein levels, and increased mTORc1 activity, as evidenced by increased p-P70S6K^T389^ (Figure 8K). We also measured the levels of ZFP36L2, P53, REDD1, and SESN2 proteins in hearts from patients with PPCM and other forms of cardiomyopathy and showed that the levels of these proteins were decreased only in PPCM samples (Figure 8K). These results suggest that alterations in the ZFP36L2/P53/REDD1/SESN2 pathway and mTORc1 activation are also potentially involved in the pathogenesis of PPCM in humans.

## Discussion

mTORc1 is regulated through protein-protein interactions to exert rapid metabolic effects in response to various stimuli, including amino acid deprivation and growth factor signaling. Despite extensive work on the regulation of mTORc1, it has never been shown to our knowledge that the mTORc1 pathway can be regulated through mRNA stability by an mRNA binding protein. We discovered a potentially novel pathway that regulates mTORc1 activity through modulation of *Mdm2* mRNA stability by ZFP36L2, resulting in altered P53 levels and subsequent REDD1 and SESN2 expression. Our findings imply the existence of another layer of mTORc1 regulation through mRNA stability of its upstream regulators in addition to generally accepted protein level regulation. Additionally, we demonstrate that, in mice, moderate modulation of mTORc1 activity determines the switch between physiological and pathophysiological cardiac hypertrophy during pregnancy, a state where proanabolic signals converge on mTORc1. This potentially novel mTORc1 regulation pathway is likely enlisted in situations that require rheostat-like regulation of mTORc1 activity and halting of unchecked activation of mTORc1.

Pregnancy is associated with a number of hemodynamic changes to ensure adequate blood supply to the mother and to the fetus for proper fetal growth and development. One prominent change during pregnancy is the growth of the LV wall and mass, which return to prepregnancy levels after delivery. Although these changes have been demonstrated in mice and humans, the molecular basis of this phenomenon is not clear. Our data demonstrate a central role for mTORc1 and P53 in this process and that the uncontrolled growth of the heart is halted by the regulation of mTORc1 activation by ZFP36L2. We also show that inadequate control of this pathway, as a result of lower expression of ZFP36L2 protein, can lead to PPCM in mice, which is partially rescued by rapamycin treatment. Because constitutive activation of mTORc1 leads to cardiomyopathy, it is not surprising that *Zfp36l2* deletion leads to a similar phenotype in pregnancy. However, this pathway plays a specific role in pregnancy because deletion of *Zfp36l2* does not exacerbate heart failure induced by TAC in mice.

Prosurvival signaling pathways are also involved in the mechanism of cardiac hypertrophy during pregnancy. Chung et al. showed that AKT and ERK1/2 are activated in the hearts of pregnant mice, while p38 is decreased (40). Additionally, mice expressing a cardiac-specific myristoylated form of AKT (activated AKT) or constitutively active glycogen synthase kinase 3β displayed an attenuated pregnancy-induced cardiac hypertrophy. They also demonstrated that progesterone increases cardiomyocyte cell size through ERK1/2 phosphorylation, and inhibition of this pathway blocked progesterone-induced hypertrophy. Because ERK1/2 and AKT can both function upstream of mTORc1, we propose that, during pregnancy, progesterone causes an increase in mTORc1 activation and cardiomyocyte hypertrophy. However, ZFP36L2 also works in parallel to keep this pathway under control and to impede unchecked activation of this process. Disruption of this regulation of mTORc1 contributes to uncontrolled cardiac growth during pregnancy and PPCM. In support of this model, we provide data showing that patients with PPCM have lower levels of ZFP36L2 protein.

A link between P53 and mTORc1 activity has been previously demonstrated. Three studies suggest that mTORc1 inhibition by genotoxic stress is dependent on P53 (36, 38, 42). A number of studies also implicated a role for P53 in mTORc1 inhibition by energy depletion (43–46); however, two other studies refuted a role for P53 in this process (47, 48). Two studies have implicated that P53 has protective effects under nonstress conditions (49, 50). Our results demonstrate that P53 plays an important role in regulating baseline mTORc1 activity through REDD1 and SESN2. Additionally, the role of P53 in cardiac physiology during pregnancy and the etiology of PPCM has not been described. Surprisingly, a significant association between PPCM and the risk of cancer was described in a cohort of 236 patients, and mutations in genes involved in DNA repair were identified in a subset of these patients (51). We show that P53 regulation of mTOR and cardiac growth during pregnancy has disease implications, as disruption of the pathway leads to mild mTORc1 hyperactivation and PPCM in mice.

We noted a reduction in ZFP36L2 protein levels and the corresponding increase in mTOR activity in the hearts of patients with PPCM (Figure 8K). Because the pathway involving ZFP36L2 regulation of mTOR activation is critical for normal cardiac growth during pregnancy and its disruption leads to PPCM in mice, alterations in ZFP36L2 levels and hyperactivation of mTORc1 potentially form part of the basis of PPCM in humans. We posit that these disruptions are triggered by the process of pregnancy. In our mouse model, the *Zfp36l2* gene is ablated from birth in mature cardiomyocytes, resulting in mTOR activation even in the absence of pregnancy, which is markedly exacerbated with the cardiac growth that occurs during pregnancy.

Our studies have not identified potential pathological mutations within the *ZFP36L2* gene in 37 patients with PPCM, suggesting that whole-body alterations of ZFP36L2 function may be pleiotropic in humans and thus evolutionarily disadvantageous. Complete deletion of the gene in mice leads to defective hematopoiesis and death within the first 2 weeks after birth (9). Two other murine models of PPCM, involving either STAT3 or PGC1α deletion in the heart (7, 52), also lack human genetic correlates to date, likely for the same reasons.

The ZFP36L2/mTOR pathway is implicated as a critical process in the development of PPCM because we were able to reduce cardiac dysfunction with rapamycin in mice. We acknowledge that PPCM is a complex and likely multifactorial disease, involving multiple pathway and endocrine processes, and that our studies do not exclude other mechanisms, such as angiogenic imbalance, which have been proposed in other mouse models (6, 7). Ultimately, these pathways may synergize as part of a multifactorial etiology for PPCM. Thus, future work combining these models will test if there is a synergistic effect on cardiac function during pregnancy to establish a model that better recapitulates human PPCM. In addition, although the results of human samples suggest the involvement of the ZFP36L2/mTOR pathway in PPCM, further studies are needed to validate the extent to which this pathway is involved in the pathogenesis of PPCM in humans with larger cohorts.

In summary, our results demonstrate the existence of a pathway that regulates mTORc1 activity through an mRNA binding protein. This pathway alters both SESN2 and REDD1 through P53 (Figure 8L). Additionally, disruption of this pathway leads to cardiac damage during the stress of pregnancy in mice, implying that inhibition of ZFP36L2-mediated regulation of mTORc1 causes context-specific effects on proanabolic processes. Our studies provide a model for understanding PPCM, and further studies are needed to validate our proposed mechanism in humans.

## Methods

### Cell culture and regents.

HEK293T cells, MEFs, and H9c2 cells were grown in Dulbecco’s Modified Eagle’s Medium (Corning) supplemented with 10% FBS (Atlanta Biologicals). HEK293T and H9c2 cells were purchased from ATCC, and *P53* KO MEFs were a gift from Nissim Hay (University of Illinois, Chicago, Illinois, USA). NRCMs were isolated as described previously (53). Mouse adult cardiomyocytes were isolated as previously reported (54) with a slight modification: our collagenase buffer contained 1.5 mg/ml collagenase 2 (Worthington) and 40 μM CaCl_2_. All cells were maintained in a 37°C incubator with 5% CO_2_ and were 70%–90% confluent when collected for analyses unless otherwise noted. To activate PI3K/Akt signaling, cells were first deprived of serum for 3 hours and then were treated with 100 nM insulin for 15 or 30 minutes. In another series of experiments, cells were pretreated with 10 nM rapamycin for 24 hours before serum deprivation. To evaluate the response of mTORC1 activity against the amino acid leucine, cells were first deprived of amino acid for 50 minutes in modified HBSS containing 25 mM glucose and 1.25 mM pyruvate and were then treated with 800 μM l-leucine (MilliporeSigma) for 10 minutes. Nutlin3 (MilliporeSigma), an inhibitor of the interaction between P53 and MDM2, was used to activate P53 at a dose of 1–10 μM for 2 to 6 hours depending on the experiment. To induce DNA damage, cells were treated with 200 μM H_2_O_2_, 20 μM etoposide, or 0.25 μM DOX for indicated duration.

### Mouse studies, survival studies after pregnancy, and rescue experiments with rapamycin.

Mice were housed in the barrier facility at Northwestern University on a 12-hour-light/dark cycle. Generation of *Zfp36l2*-ﬂoxed (*Zfp36l2*^ﬂ/ﬂ^) mice has been described previously (27). *Zfp36l2*^fl/fl^ mice were crossed with αMHC-Cre mice to generate *Zfp36l2*^fl/fl^; αMHC-Cre mice, which were then mated with *Zfp36l2*^fl/fl^ mice to generate cardiac-specific *Zfp36l2-*KO mice and floxed control littermates for experiments. αMHC-Cre mice and their nontransgenic littermates were also included as controls. Female mice were bred starting at 8 weeks of age. Pregnant KO mice were treated with rapamycin (1 mg/kg) or vehicle by subcutaneous injections every other day from day 12 of pregnancy until delivery. Nonpregnant mice were treated with the same amount of rapamycin or vehicle by subcutaneous injections every other day for 9 days starting from 9 weeks of age. Stock solution of rapamycin (20 mg/mL in ethanol) was suspended in 5% Tween 80, 5% PEG 400 dissolved in PBS for each injection.

### Coronary ligation and TAC.

The surgical protocol was performed as previously described (55). Briefly, mice were anesthetized with isoflurane, with induction at 3% and maintenance at 1.5%–2%. The animals were placed in a supine position, and ECG leads were attached. Body temperature was monitored using a rectal probe and was maintained at 37°C with heating pads throughout the experiment. A catheter was inserted into the trachea and was then attached to the mouse ventilator via a Y-shaped connector. The mice were ventilated at a tidal volume of 200 μL and a rate of 105 breaths/min using a rodent ventilator. The chest was then opened by an incision in the left fourth intercostal space. The left anterior descending artery was occluded with an 8-0 silk suture. Ischemia was confirmed by pallor of the anterior wall of the left ventricle and by ST-segment elevation and QRS widening on the ECG. After confirmation of ligation, the chest was closed in layers. The mice were kept warm with heating pads and on 100% oxygen via nasal cannula. Animals were given buprenorphine for postoperative pain. Chronic pressure overload was induced by TAC in mice at 10–12 weeks as described previously (56).

### In vitro [^35^S]-methionine incorporation assay.

Cells were first deprived of amino acid for 50 minutes in HBSS and then incubated with complete medium containing [^35^S]-methionine (0.01 mCi/mL; Perkin-Elmer). After 1 hour, cells were lysed with RIPA buffer, and protein concentration was determined. Proteins were precipitated with trichloroacetic acid (10%) at 4°C overnight and washed with acetone, and radioactivity was determined by liquid scintillation counting. Radioactive levels were normalized to protein concentration.

### Reverse transcription and quantitative real-time PCR.

RNA was isolated from cells or heart samples using RNA-STAT60 (Tel-Test) and reverse transcribed with TaqMan Reverse Transcription Reagents (Invitrogen) according to the manufacturers’ instructions. Quantification of relative gene expression was done using Fast SYBR Green Master Mix (Applied Biosystems) and run on 7500 Fast Real-Time PCR system (Applied Biosystems). The relative gene expression was determined using differences in Ct values between the gene of interest and housekeeping control genes, which were *Actb*, *Hprt1*, and/or *18S*, depending on the experiment.

### mRNA stability assay and RNA co-IP.

Cells were treated with 7.5 μM actinomycin D (MilliporeSigma) for indicated times, and RNA was harvested and processed as described above. RNA Co-IP experiments were conducted as described previously (57).

### Transfection.

siRNAs were purchased from Dharmacon. siRNAs were transfected using Dharmafect 1 Transfection Reagent (Dharmacon). Plasmids were transfected using Lipofectamine 2000 reagent (Invitrogen), Lipofectamine 3000 (Invitrogen), or calcium phosphate transfection. Experiments were performed 24~48 hours after transfection. LAMP1-mGFP, which was used to express human LAMP1, was a gift from Esteban Dell’Angelica (Addgene plasmid 34831).

### Western blotting.

Proteins were resolved on 4%–12% Novex Bis-Tris poly-acrylamide gel (Invitrogen) and blotted onto nitrocellulose membrane (Invitrogen). Membranes were incubated with the following primary antibodies overnight, before the addition of HRP-conjugated secondary antibodies. P-P70S6K (T389), P70S6K, P-S6 (S240/244), S6, P-AKT (S473), AKT, P-AMPK (T172), AMPK, TSC2, P53 (1C12), Ubiquitin, P-H2AX, and GAPDH were from Cell Signaling Technology. HPRT-1, REDD1, SESN2, and MDM2 were from Proteintech. ZFP36L2 was obtained from Abcam, MilliporeSigma, and Santa Cruz Biotechnology. The presence of target protein was visualized using Super Surgical Western Pico ECL substrate (Pierce). Quantification of Western blotting images was done using ImageJ (NIH). Antibody suppliers and catalog numbers are shown in Supplemental Table 4.

### P53 immunoprecipitation.

After pretreatment with 20 μM MG132 for 5 hours, cells were lysed in buffer containing 50 mM Tris-HCl (pH 7.5), 300 mM NaCl, 0.5% Triton-X, and 10 mM N-ethylmaleimide. Precleared cell lysates (500 μg) were incubated with anti-P53 antibody or control IgG in lysis buffer at 4°C overnight with rotation. Antibody-protein complexes were collected with Protein G-Agarose beads (MilliporeSigma) and washed with ice-cold immunoprecipitation buffer according to the manufacturer’s instructions. Immunoprecipitates were subjected to Western blotting to probe ubiquitination of P53.

### Protein stability assay.

Endogenous P53 protein degradation was analyzed by a protein stability assay. Briefly, H9c2 cells or NRCMs transfected with *Zfp36l2* siRNA for 48 hours were incubated with 50 μg/ml cycloheximide (MilliporeSigma) for 30 minutes. P53 protein levels were analyzed by Western blotting. The results from Western blot analysis were quantified by densitometry and normalized against the 0-minute time point.

### Cell death analysis.

Cells were stained with 5 μg/ml PI and in HBSS (Life Technologies) and Hoechst 33342 (Invitrogen) for nuclei counter stain. Images were acquired on a Zeiss AxioObserver.Z1 microscope, and cell death was defined as the number of PI-positive nuclei over total number of nuclei.

### ROS measurements.

Intracellular ROS levels were monitored by dichlorofluorescein (DCF) fluorescence (Invitrogen). After pretreatment with vehicle or 600 μM H_2_O_2_ for 90 minutes, cells were incubated in HBSS containing 1 μM DCF for 10 minutes according to the manufacturer’s protocol. Cells were washed and stained with Hoechst 33342 for nuclei counter stain. DCF fluorescence was recorded by a fluorescent microscope.

### RNA-Seq experiments.

H9c2 cells were cultured as above and then transfected with either control or *Zfp36l2* siRNA (a mixture of 4 siRNAs for *Zfp36l2*, the target sequences of which are GCAAGUACGGCGAGAAGUG, CAAAUCAACUCCACGCGCU, UGUCAGCUUUCUACGAUAU, and GCGCUGGUCAACAAGGAAA) using Dharmafect 1 Transfection Reagent (Dharmacon). The media were replaced after 24 hours, and then the cells were harvested 48 hours after transfection. Total RNA was extracted using NucleoSpin (Takara Bio) per the manufacturer’s instructions. RNA quality was tested using a Nanodrop 2000 spectrophotometer (Thermo Fisher Scientific). Six RNA samples each of control and Zfp36l2 KD cells were submitted to the NU-Seq Core facility for library preparation and sequencing. Libraries were prepared using the TruSeq mRNA-Seq Library Prep Kit (Illumina) per the manufacturer’s instructions, with each sample producing 50-bp single-end reads and given 1 index sequence. All samples were sequenced using 1 lane on a HiSeq 4000 (Illumina) and processed to FASTQ files using standard base-calling software and parameters.

### RNA-Seq data analysis.

The raw FASTQ files were used as input for kallisto (version 0.44.0) (58) to quantify transcript-level expression using the flags “–bias –b 100.” This analysis used the annotations for the *Rattus norvegicus* transcriptome from Ensembl version 93 (July 2018). Next, the estimated expressions were used as input for *sleuth-CN* (https://github.com/warrenmcg/sleuth-CN, master branch, commit 461b95b), an extension of the *sleuth* package (59) that uses compositional data analysis and allows for the choice of “reference features” for normalization, similar to qPCR (60). When there is an expectation of a large number of features changing, such as in this study, this approach provides a more accurate interpretation of which features are changing and in what direction(60). The expression values were normalized to *Actb* and *Hprt1*, which have been used as reference genes or expression markers in previous human and rodent cardiomyocyte studies (61, 62). The transcripts per million were modeled for sleuth’s differential analysis using default settings, and the Wald statistic was used to test for differential expression at the transcript level, looking for differences between the *Zfp36l2*-KD and control groups. In this data set, absolute β values for the selected reference genes were less than 0.1, and their variance (“sigma squared”) was less than 0.1, indicating that their normalized expression values were relatively consistent across samples and with each other.

Then, from the transcript-level differential analysis, we produced a gene-level differential analysis using the *P* value aggregation method developed by Yi et al. (63). This method does not attempt to estimate gene-level expression differences, but rather tests whether any isoform of a particular gene has changed, with a significant *P* value indicating that at least 1 isoform of that change has indeed changed. Genes were considered significantly regulated if the adjusted *P* value after aggregation was equal to or less than 0.05. From these results, we added 2 columns: “number of sig. transcripts,” which showed how many differentially expressed isoforms were present for that gene, as well as “direction of sig. transcripts,” which showed whether the DE isoforms had a concordant change (all “up” or “down”) or discordant (“mixed”) change.

To generate a heatmap, the sleuth-CN–normalized transcript expression values were first transformed to *z* scores (subtract from each expression value the mean expression value across samples and then divide by the standard deviation across samples). The transformed expression values are depicted on a scale from blue (most downregulated among samples) to red (most upregulated among samples), and the samples are annotated by condition (blue for KD samples; gray for control samples). Both the samples (columns) and transcripts (rows) were hierarchically clustered, with the clustering results depicted by the dendrograms. The samples cluster by condition, and the transcripts broadly cluster by upregulated transcripts and downregulated transcripts.

The volcano plots and heatmap plots were created using the standard plotting functions with sleuth; the ggrepel package (https://slowkow.com/ggrepel) was used for labeling the volcano plot. All of the sleuth-CN analysis and plotting was performed in R, version 3.5.1, and the pipeline for analyzing the data from start to finish was done using bioconda and snakemake. The full pipeline can be found on GitHub (https://github.com/warrenmcg/Zfp36l2_paper_analysis, master branch, commit 4294996).

### Pathway analysis.

Pathway analysis was performed using the DAVID Bioinformatics Database (v6.8; https://david.ncifcrf.gov) with the top 10% of significantly altered transcripts with the greatest absolute log_2_ fold change as input.

### In silico analysis of AREs.

AREs were identified by either of the following two criteria: (a) curated ARE entry in the AREsite Database (64) or (b) presence of AUUUA sequence in the 3′-UTR region of reference mRNA sequences by manual search.

### Echocardiography, organ harvest, and histological analysis.

Parasternal short- and long-axis views of the heart were obtained using a Vevo 770 high-resolution imaging system with a 30 MHz scan head. 2D and M-mode images were obtained and analyzed. Cardiac function was calculated as previously described (65). At the time of tissue harvest, mice were anesthetized with 250 mg/kg dose of freshly prepared Tribromoethanol (Avertin). Tissue was excised, rinsed in PBS to remove excess blood, and was then freshly frozen in liquid nitrogen and stored at –80°C. For histological analysis, immediately after making a small incision in the inferior vena cava, beating heart was perfused with relaxing buffer (100 mM KCl, 5 mM EGTA, and 5 mM Na pyrophosphate) until termination of beating, followed immediately with 30 ml of 4% paraformaldehyde using the peristaltic pump (7 mL/min) from the apex of left ventricle through a 22-gauge needle. The excised heart tissue was further fixed overnight in 4% paraformaldehyde before graded dehydration in 70%, 80%, 90%, and 100% ethanol. The tissue sample was further dehydrated with xylene and embedded into paraffin. Sections were stained with hematoxylin and eosin for evaluation of general cardiac morphology and tissue organization. Masson’s trichrome staining was used to visualize cardiac fibrosis.

### Immunohistochemistry.

Cells grown on a cover glass were fixed with 4% paraformaldehyde for 15 minutes. The cells were then washed with PBS, blocked with 5% goat serum and 0.1% Triton X-100 in PBS for 60 minutes, and incubated overnight with anti–phospho-Histone H2A.X antibody (Ser139; Cell Signaling Technology) or anti-TSC2 antibody (Cell Signaling Technology) in blocking buffer. The bound antibodies were labeled with an Alexa Fluor(R) 488 anti-rabbit secondary antibody or an Alexa Fluor(R) 594 anti-rabbit secondary antibody, and nuclei were stained with DAPI. The cover glass was mounted on a slide glass and was visualized using a fluorescent microscopy.

### Human samples.

All donors and transplant hearts received in situ cold cardioplegia to protect the myocardium. The control participants and patients with PPCM were age, sex, and race matched. Their medications are included in Supplemental Table 3. All donors had LV mass indexes of less than 95 g/m^2^, consistent with MESA criteria (66). Heart weights were well below any pathologic level, as defined in the literature (67, 68). The control donors and donors with PPCM were not statistically different in age, height, weight, BMI, BSA, and the majority of echo parameters, except for LVEF.

### Sequencing in the Zfp36l2 gene in patients with PPCM.

The extracted DNA samples from patients with PPCM were obtained in-house. We performed PCR with the DNA samples using primers flanking the *Zfp36l2* gene, followed by whole-exon sequencing of *Zfp36l2* with the Sanger sequencing technique. Variation in the sequence was determined by aligning the sequence of each sample with *Zfp36l2*. Allele frequency in the variants was searched on the GnomAD website (https://gnomad.broadinstitute.org/).

### Statistics.

Data are presented as mean ± SEM. Single comparisons were assessed by an unpaired Student’s 2-tailed *t* test. Statistical significance was assessed with ANOVA, with post hoc Tukey’s test for multiple group comparison; a 1-way ANOVA was performed when the data set had 1 independent variable, while a 2-way ANOVA was done with 2 independent variables. Log-rank test was used for analysis of survival curves, with Bonferroni’s post hoc analyses for multiple group comparison. A *P* value of less than 0.05 was considered statistically significant. The analysis was conducted using GraphPad Prism9 software.

### Study approval.

All animal studies were approved by the Institutional Animal Care and Use Committee at Northwestern University and were performed in accordance with guidelines from the NIH. Nonfailing and PPCM human heart samples were obtained from the University of Pennsylvania (IRB approval 848421). Failing human hearts (non-PPCM samples) were provided by the Clinical Trials Unit, Bluhm Cardiovascular Institute at Northwestern Memorial Hospital (Chicago, Illinois, USA) (IRB approval IRB STU00012288). Informed consent was obtained from all the transplant patients and from the families of the organ donors before tissue collection. Protocols for tissue procurement were approved by the University of Pennsylvania and Northwestern University, which is AAHRPP accredited.

## Author contributions

HK designed, performed, and analyzed Western blotting and immunoprecipitation experiments, qPCR experiments, in vitro radioactive amino acid incorporation assays, mRNA and protein stability assays, RNA co-IP experiments, cell death and ROS measurement assays, immunocytochemistry and mouse studies; performed cardiomyocyte isolation and RNA-Seq experiments; bred the mouse colonies; and drafted the manuscript. YT performed and analyzed Western blotting experiments, qPCR experiments, immunocytochemistry, and mouse studies. HCC, JSS, and WAM analyzed RNA-Seq experiments and performed pathway analyses. ADJ assisted with in silico analyses of AREs and organ harvest from mice. IBS provided critical expert guidance on the manuscript. CC managed the mouse colonies. ZA, JL, and PJB provided reagents. HK, YT, and HA wrote the manuscript, and HK, YT, JSS, and HA edited the final version. HA conceived and supervised the study and drafted the manuscript. All authors discussed the results and critically reviewed the manuscript.

## Supplementary Material

Supplemental data

Supplemental table 1

Supplemental table 2

Supplemental table 3

Supplemental table 4

## Figures and Tables

**Table 1:**
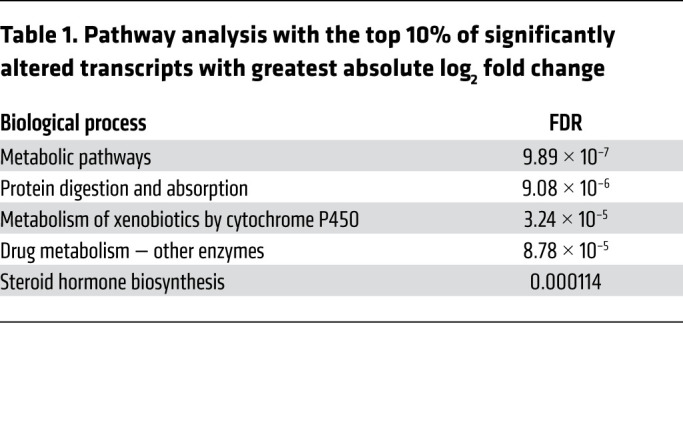
Pathway analysis with the top 10% of significantly altered transcripts with greatest absolute log_2_ fold change

## Version 1 03/22/2022

In-Press Preview

## Version 2 05/16/2022

Electronic publication

## References

[1] Sanghavi M, Rutherford JD (2014). Cardiovascular physiology of pregnancy. Circulation.

[2] Robson SC (1987). Haemodynamic changes during the puerperium: a Doppler and M-mode echocardiographic study. Br J Obstet Gynaecol.

[3] Ardehali H (2003). Peripartum cardiomyopathy. Minerva Cardioangiol.

[4] Lindley KJ (2019). Peripartum cardiomyopathy: progress in understanding the etiology, management, and prognosis. Heart Fail Clin.

[5] Patel PA (2017). A contemporary review of peripartum cardiomyopathy. Clin Med (Lond).

[6] Goland S (2016). Angiogenic imbalance and residual myocardial injury in recovered peripartum cardiomyopathy patients. Circ Heart Fail.

[7] Patten IS (2012). Cardiac angiogenic imbalance leads to peripartum cardiomyopathy. Nature.

[8] Brooks SA, Blackshear PJ (2013). Tristetraprolin (TTP): interactions with mRNA and proteins, and current thoughts on mechanisms of action. Biochim Biophys Acta.

[9] Stumpo DJ (2009). Targeted disruption of Zfp36l2, encoding a CCCH tandem zinc finger RNA-binding protein, results in defective hematopoiesis. Blood.

[10] Ramos SB (2012). Characterization of DeltaN-Zfp36l2 mutant associated with arrest of early embryonic development and female infertility. J Biol Chem.

[11] Hodson DJ (2010). Deletion of the RNA-binding proteins ZFP36L1 and ZFP36L2 leads to perturbed thymic development and T lymphoblastic leukemia. Nat Immunol.

[12] Galloway A (2016). RNA-binding proteins ZFP36L1 and ZFP36L2 promote cell quiescence. Science.

[13] Saxton RA, Sabatini DM (2017). mTOR signaling in growth, metabolism, and disease. Cell.

[14] Meikle L (2005). A mouse model of cardiac rhabdomyoma generated by loss of Tsc1 in ventricular myocytes. Hum Mol Genet.

[15] Taneike M (2016). mTOR hyperactivation by ablation of tuberous sclerosis complex 2 in the mouse heart induces cardiac dysfunction with the increased number of small mitochondria mediated through the down-regulation of autophagy. PLoS One.

[16] Peng M (2014). Sestrins function as guanine nucleotide dissociation inhibitors for Rag GTPases to control mTORC1 signaling. Cell.

[17] Zhu Y (2013). Mechanistic target of rapamycin (Mtor) is essential for murine embryonic heart development and growth. PLoS One.

[18] Mazelin L (2016). mTOR inactivation in myocardium from infant mice rapidly leads to dilated cardiomyopathy due to translation defects and p53/JNK-mediated apoptosis. J Mol Cell Cardiol.

[19] Zhang D (2010). MTORC1 regulates cardiac function and myocyte survival through 4E-BP1 inhibition in mice. J Clin Invest.

[20] Harrison DE (2009). Rapamycin fed late in life extends lifespan in genetically heterogeneous mice. Nature.

[21] Flynn JM (2013). Late-life rapamycin treatment reverses age-related heart dysfunction. Aging Cell.

[22] McMullen JR (2004). Inhibition of mTOR signaling with rapamycin regresses established cardiac hypertrophy induced by pressure overload. Circulation.

[23] Bell SE (2006). The RNA binding protein Zfp36l1 is required for normal vascularisation and post-transcriptionally regulates VEGF expression. Dev Dyn.

[24] Stumpo DJ (2004). Chorioallantoic fusion defects and embryonic lethality resulting from disruption of Zfp36L1, a gene encoding a CCCH tandem zinc finger protein of the Tristetraprolin family. Mol Cell Biol.

[25] Sato T (2018). mRNA-binding protein tristetraprolin is essential for cardiac response to iron deficiency by regulating mitochondrial function. Proc Natl Acad Sci U S A.

[26] Sanduja S (2011). The roles of TTP and BRF proteins in regulated mRNA decay. Wiley Interdiscip Rev RNA.

[27] Dumdie JN (2018). Chromatin modification and global transcriptional silencing in the oocyte mediated by the mRNA decay activator ZFP36L2. Dev Cell.

[28] Zhang L (2013). ZFP36L2 is required for self-renewal of early burst-forming unit erythroid progenitors. Nature.

[29] Dibble CC, Manning BD (2013). Signal integration by mTORC1 coordinates nutrient input with biosynthetic output. Nat Cell Biol.

[30] Menon S (2014). Spatial control of the TSC complex integrates insulin and nutrient regulation of mTORC1 at the lysosome. Cell.

[31] Bar-Peled L (2013). A tumor suppressor complex with GAP activity for the Rag GTPases that signal amino acid sufficiency to mTORC1. Science.

[32] Wolfson RL (2016). Sestrin2 is a leucine sensor for the mTORC1 pathway. Science.

[33] Canal M (2014). RTP801/REDD1: a stress coping regulator that turns into a troublemaker in neurodegenerative disorders. Front Cell Neurosci.

[34] Parmigiani A, Budanov AV (2016). Sensing the environment through sestrins: implications for cellular metabolism. Int Rev Cell Mol Biol.

[35] Vousden KH, Prives C (2009). Blinded by the light: the growing complexity of p53. Cell.

[36] Budanov AV, Karin M (2008). p53 target genes sestrin1 and sestrin2 connect genotoxic stress and mTOR signaling. Cell.

[37] Budanov AV (2002). Identification of a novel stress-responsive gene Hi95 involved in regulation of cell viability. Oncogene.

[38] Ellisen LW (2002). REDD1, a developmentally regulated transcriptional target of p63 and p53, links p63 to regulation of reactive oxygen species. Mol Cell.

[39] Michael D, Oren M (2003). The p53-Mdm2 module and the ubiquitin system. Semin Cancer Biol.

[40] Chung E (2012). Akt and MAPK signaling mediate pregnancy-induced cardiac adaptation. J Appl Physiol (1985).

[41] Hennig M (2017). Prenatal mechanistic target of rapamycin complex 1 (m TORC1) inhibition by rapamycin treatment of pregnant mice causes intrauterine growth restriction and alters postnatal cardiac growth, morphology, and function. J Am Heart Assoc.

[42] Feng Z (2005). The coordinate regulation of the p53 and mTOR pathways in cells. Proc Natl Acad Sci U S A.

[43] Ben Sahra I (2011). Metformin, independent of AMPK, induces mTOR inhibition and cell-cycle arrest through REDD1. Cancer Res.

[44] Jones RG (2005). AMP-activated protein kinase induces a p53-dependent metabolic checkpoint. Mol Cell.

[45] Levine AJ (2006). Coordination and communication between the p53 and IGF-1-AKT-TOR signal transduction pathways. Genes Dev.

[46] Sofer A (2005). Regulation of mTOR and cell growth in response to energy stress by REDD1. Mol Cell Biol.

[47] Ben-Sahra I (2013). Sestrin2 integrates Akt and mTOR signaling to protect cells against energetic stress-induced death. Cell Death Differ.

[48] Brugarolas J (2004). Regulation of mTOR function in response to hypoxia by REDD1 and the TSC1/TSC2 tumor suppressor complex. Genes Dev.

[49] Mak TW (2017). p53 regulates the cardiac transcriptome. Proc Natl Acad Sci U S A.

[50] Sablina AA (2005). The antioxidant function of the p53 tumor suppressor. Nat Med.

[51] Pfeffer TJ (2019). Increased cancer prevalence in peripartum cardiomyopathy. JACC CardioOncol.

[52] Hilfiker-Kleiner D (2007). A cathepsin D-cleaved 16 kDa form of prolactin mediates postpartum cardiomyopathy. Cell.

[53] Ardehali H (2005). Cardioprotective role of the mitochondrial ATP-binding cassette protein 1. Circ Res.

[54] Ackers-Johnson M (2016). A simplified, Langendorff-free method for concomitant isolation of viable cardiac myocytes and nonmyocytes from the adult mouse heart. Circ Res.

[55] Sawicki KT (2015). Increased heme levels in the heart lead to exacerbated ischemic injury. J Am Heart Assoc.

[56] Wu R (2012). Hexokinase II knockdown results in exaggerated cardiac hypertrophy via increased ROS production. EMBO Mol Med.

[57] Bayeva M (2012). mTOR regulates cellular iron homeostasis through tristetraprolin. Cell Metab.

[58] Bray NL (2016). Near-optimal probabilistic RNA-seq quantification. Nat Biotechnol.

[59] Pimentel H (2017). Differential analysis of RNA-seq incorporating quantification uncertainty. Nat Methods.

[61] Vesentini N (2012). Selection of reference genes in different myocardial regions of an in vivo ischemia/reperfusion rat model for normalization of antioxidant gene expression. BMC Res Notes.

[62] Brattelid T (2010). Reference gene alternatives to Gapdh in rodent and human heart failure gene expression studies. BMC Mol Biol.

[63] Yi L (2018). Gene-level differential analysis at transcript-level resolution. Genome Biol.

[64] Gruber AR (2011). AREsite: a database for the comprehensive investigation of AU-rich elements. Nucleic Acids Res.

[65] Ichikawa Y (2014). Cardiotoxicity of doxorubicin is mediated through mitochondrial iron accumulation. J Clin Invest.

[66] Brumback LC (2010). Body size adjustments for left ventricular mass by cardiovascular magnetic resonance and their impact on left ventricular hypertrophy classification. Int J Cardiovasc Imaging.

[67] De la Grandmaison GL (2001). Organ weight in 684 adult autopsies: new tables for a Caucasoid population. Forensic Sci Int.

[68] Kumar NT (2014). Postmortem heart weight: relation to body size and effects of cardiovascular disease and cancer. Cardiovasc Pathol.

