# Supplementary material for: ZFP36L2 suppresses mTORc1 through a P53-dependent pathway to prevent peripartum cardiomyopathy in mice
Source: J Clin Invest. 2022 May 16;132(10):e154491. doi: 10.1172/JCI154491 (PMC9106345; doi:10.1172/JCI154491)
Supplement: Supplemental data [file jci-132-154491-s143.pdf]

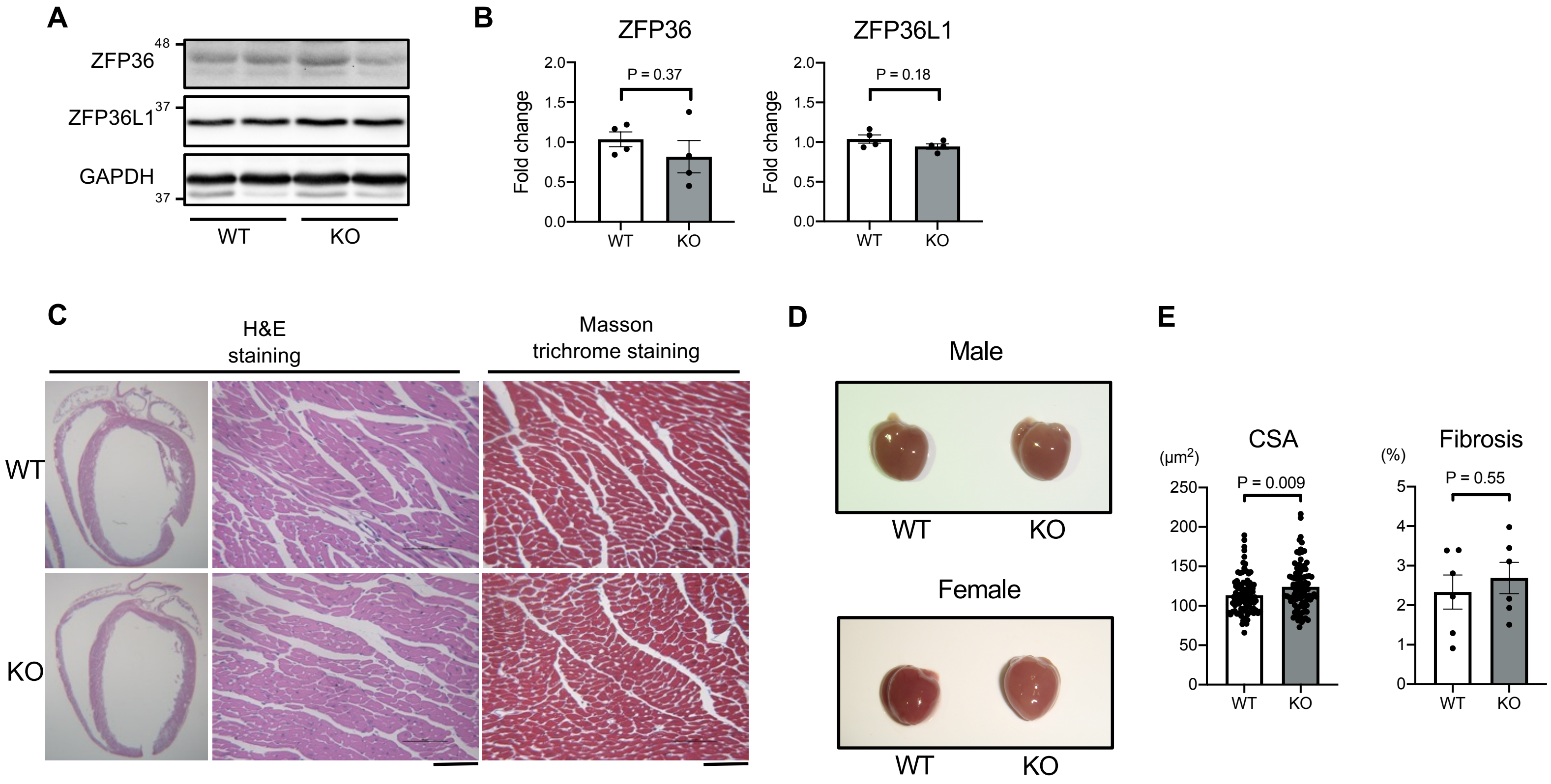

Supplemental Figure 1. Cardiac characterization of *Zfp36l2* KO mice at baseline.
(A,B) TTP (ZFP36) and ZFP36L1 protein levels in the hearts of mice with *Zfp36l2* deletion (*n* = 4). **(C)** Histology of homozygous *Zfp36l2* KO and control littermates. The scale indicates 50μm. **(D)** Gross examination of the hearts of *Zfp36l2* KO and littermate controls. **(E)** Cross sectional area and degree of fibrosis in *Zfp36l2* KO and littermate controls (*n* = 3 mice for both panels and 30 individual cardiomyocytes for CSA and 2 sections per mouse for fibrosis). Data were analyzed by unpaired Student *t* test.

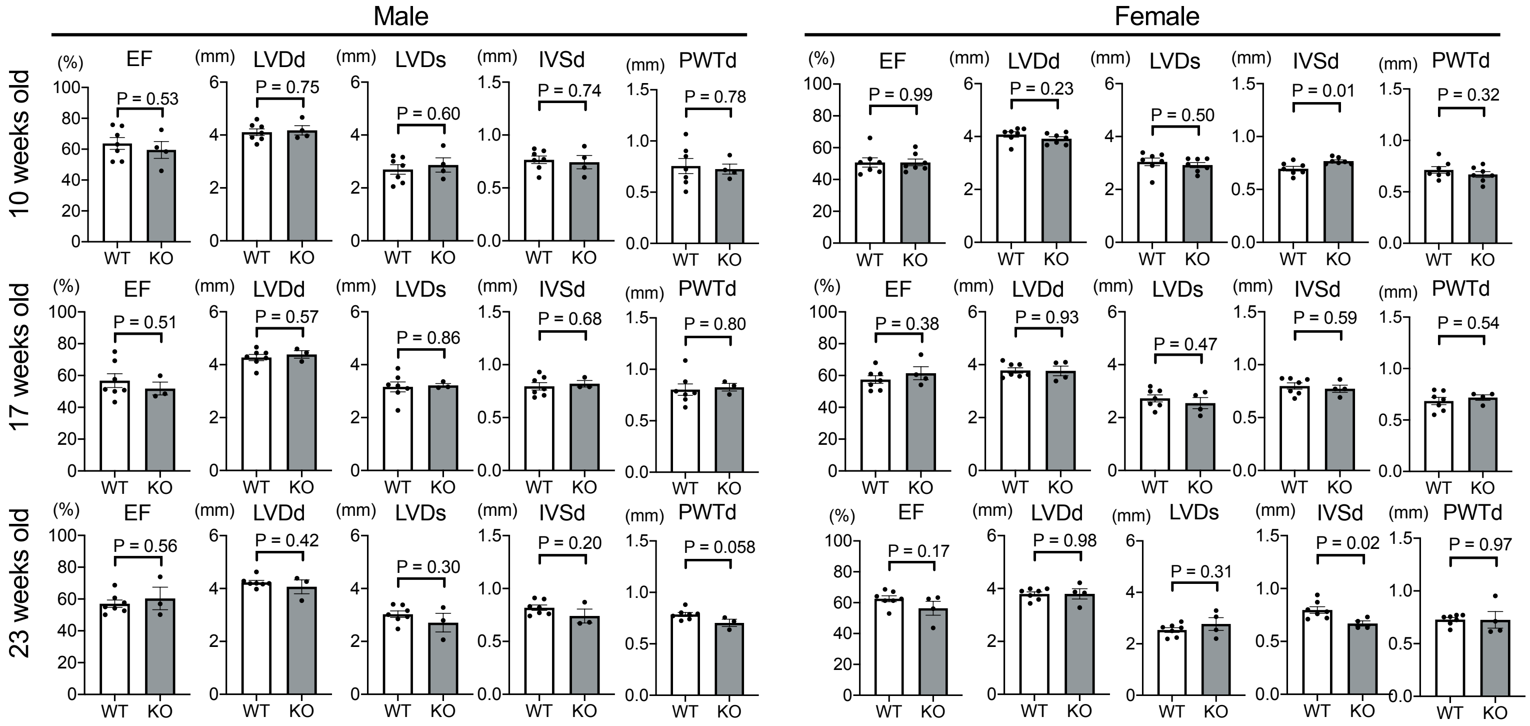

Supplemental Figure 2. Data in echocardiography of male and female *Zfp36l2* KO mice and their control littermates at baseline at 10, 17, and 23 week of age (n = 3-7). Data were analyzed by unpaired Student t test.

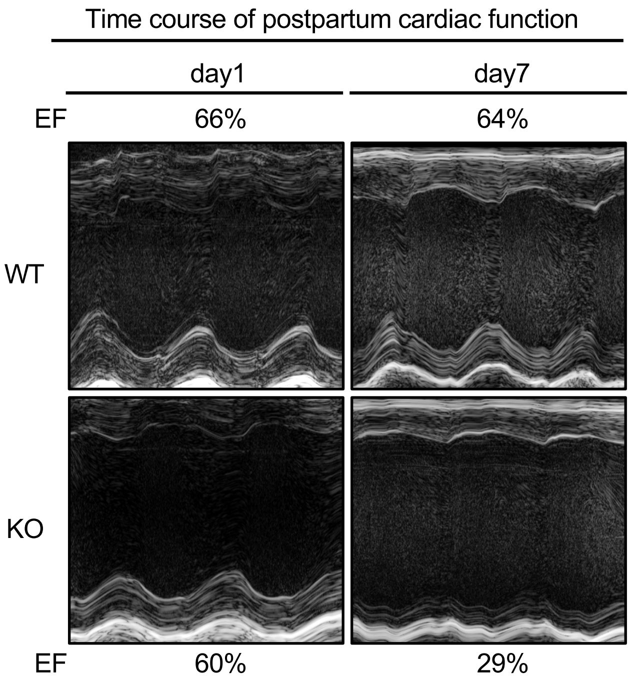

Supplemental Figure 3. Time course of postpartum cardiac dysfunction in *Zfp36l2* KO mice, illustrating cardiomyopathy 7 days after delivery.

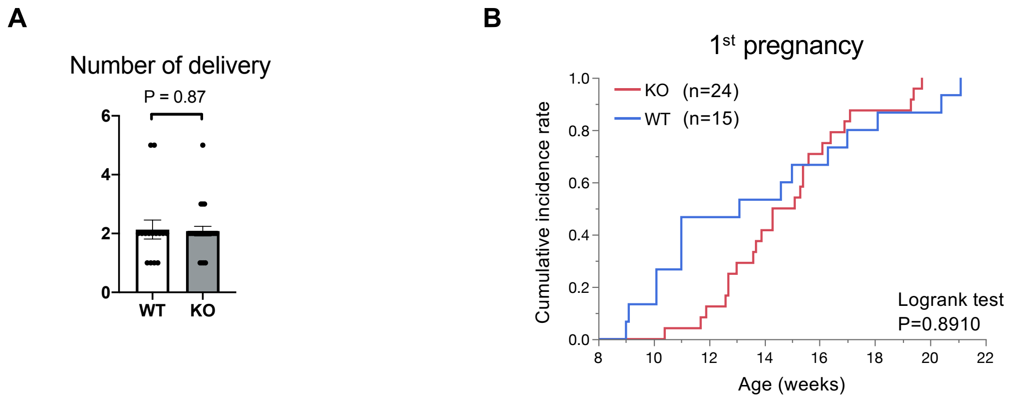

Supplemental Figure 4. pregnancy of *Zfp36l2* KO mice.
(A) Number of deliveries in the control and KO mice (*n* = 15-24). **(B)** Time to the first delivery in control and KO mice. Data were analyzed by unpaired Student t test (A) or Log-rank test (B).

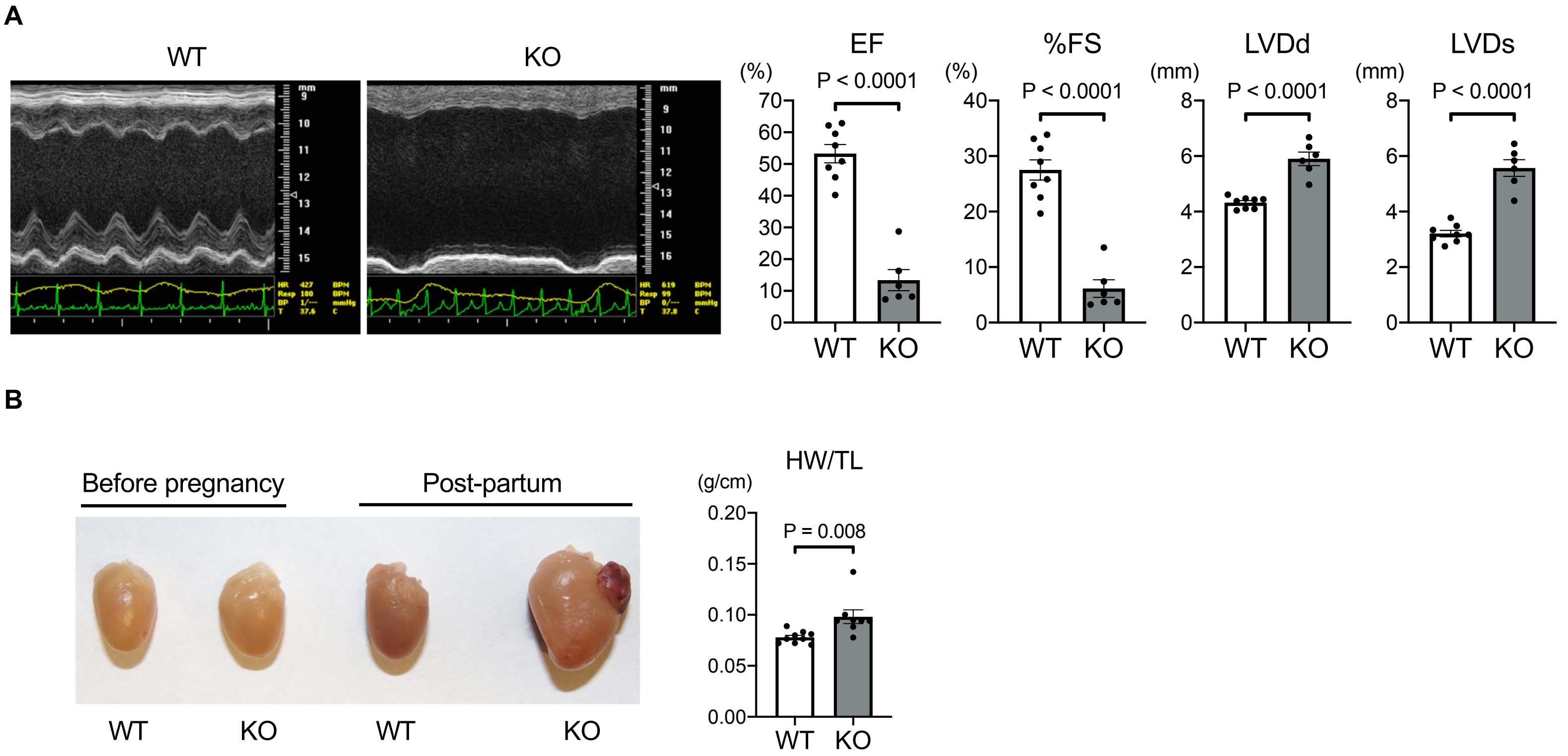

Supplemental Figure 5. Cardiac characterization of *Zfp36l2* KO female mice after second pregnancy.
(A) Echo parameters, including EF, FS, LVDd, and LVDs in *Zfp36l2* KO mice and their littermate controls after second pregnancy (*n* = 6-8). **(B)** Gross examination and HW/TL of *Zfp36l2* KO and their littermate controls after second pregnancy (*n* = 8-9). Data were analyzed by unpaired Student *t* test.

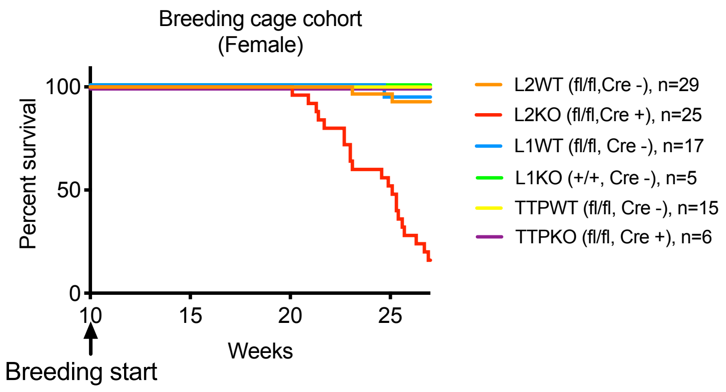

Supplemental Figure 6. **Survival of mice with cardiac specific deletion of *Zfp36*, *Zfp36l1* and *Zfp36l2* up to 27 weeks after breeding.** TTP refers to *Zfp36*, L1 refers to *Zfp36l1* and L2 refers to *Zfp36l2*.

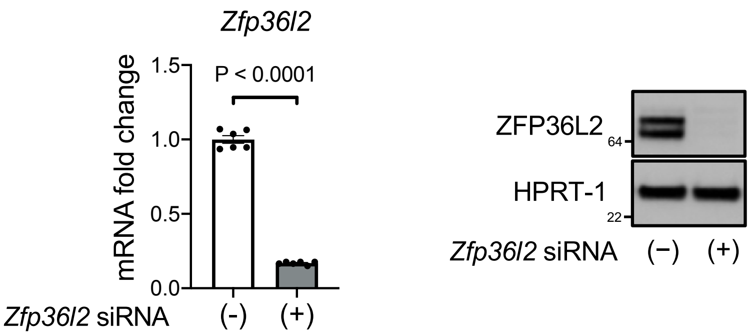

Supplemental Figure 7. Effective reduction in *Zfp36l2* mRNA (left, *n* = 6) and protein (right) levels in H9c2 cells after treatment with *Zfp36l2* siRNA. Data were analyzed by unpaired Student *t* test.

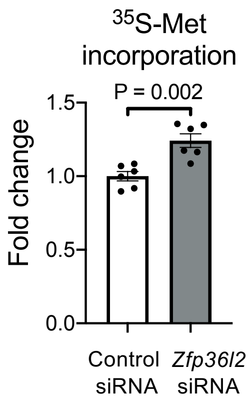

Supplemental Figure 8. ^{35}S methionine incorporation into proteins in H9c2 cells treated with control of *Zfp36l2* siRNA (*n* = 6). Data were analyzed by unpaired Student *t* test.

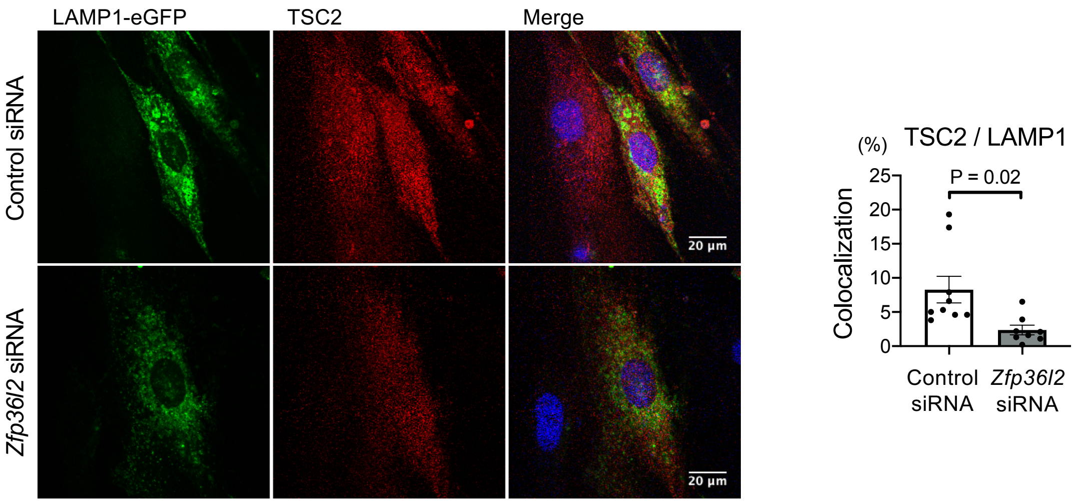

Supplemental Figure 9. TSC2 localization to lysosome in *Zfp36l2* KD cells. A LAMP-GFP construct was transfected into H9c2 cells in the presence and absence of *Zfp36l2* siRNA, and were stained with TSC2 antibody. A summary of 8-9 fields is shown in the bar graph next to the image. Data were analyzed by unpaired Student *t* test.

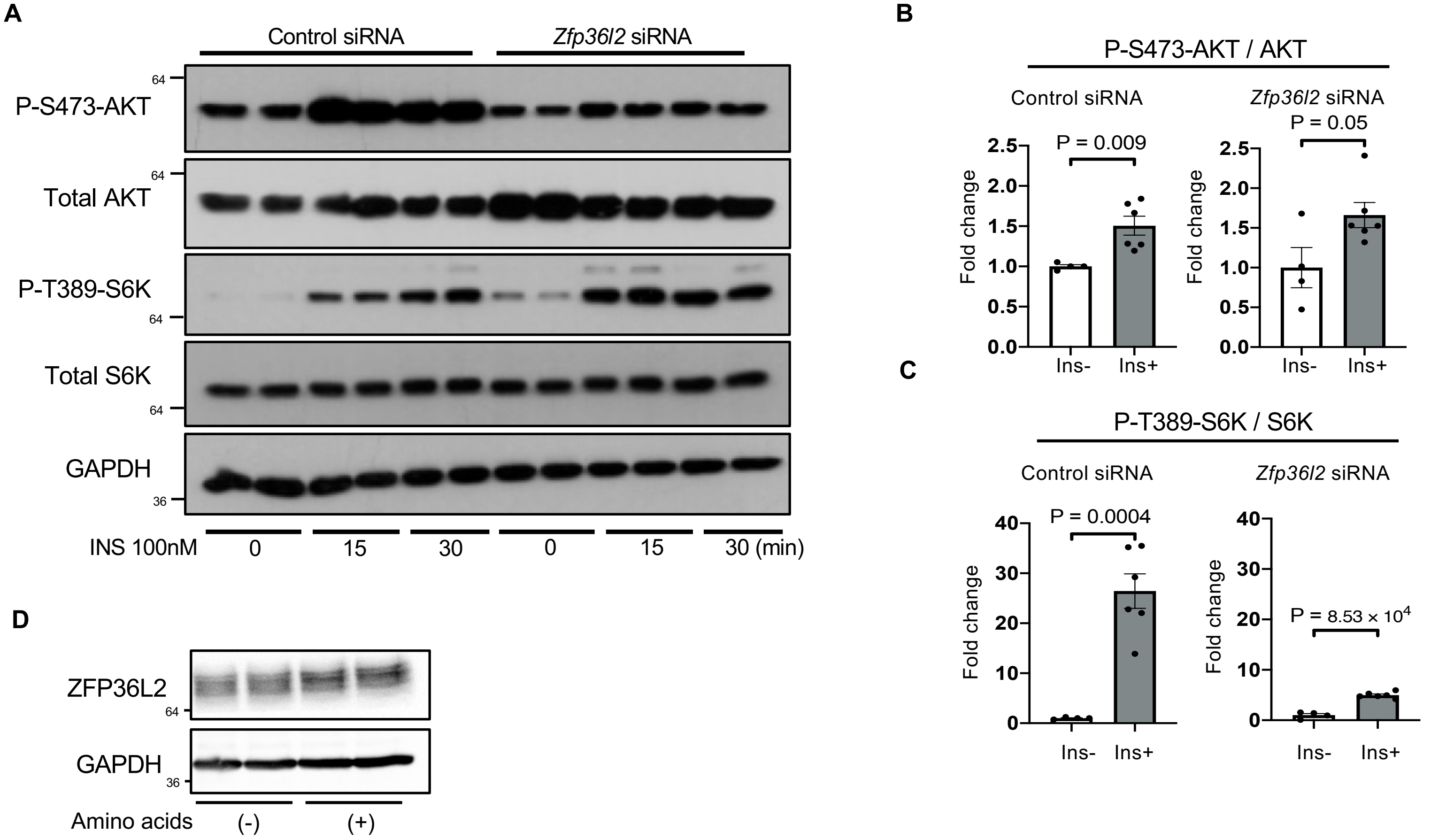

Supplemental Figure 10. P70S6K^{T389} and AKT^{S473} phosphorylation after insulin stimulation in control and *Zfp36l2* siRNA treated H9c2 cells.
(A) Western blot image of extracts of H9c2 cells after different treatments. **(B)** Summary bar graph of AKT^{S473} phosphorylation over total AKT (n = 4-6). **(C)** Summary bar graph of P70S6K^{T389}phosphorylation over total S6K (n = 4-6). **(D)** Regulation of ZFP36L2 by amino acid starvation and supplementation. Data were analyzed by unpaired Student *t* test.

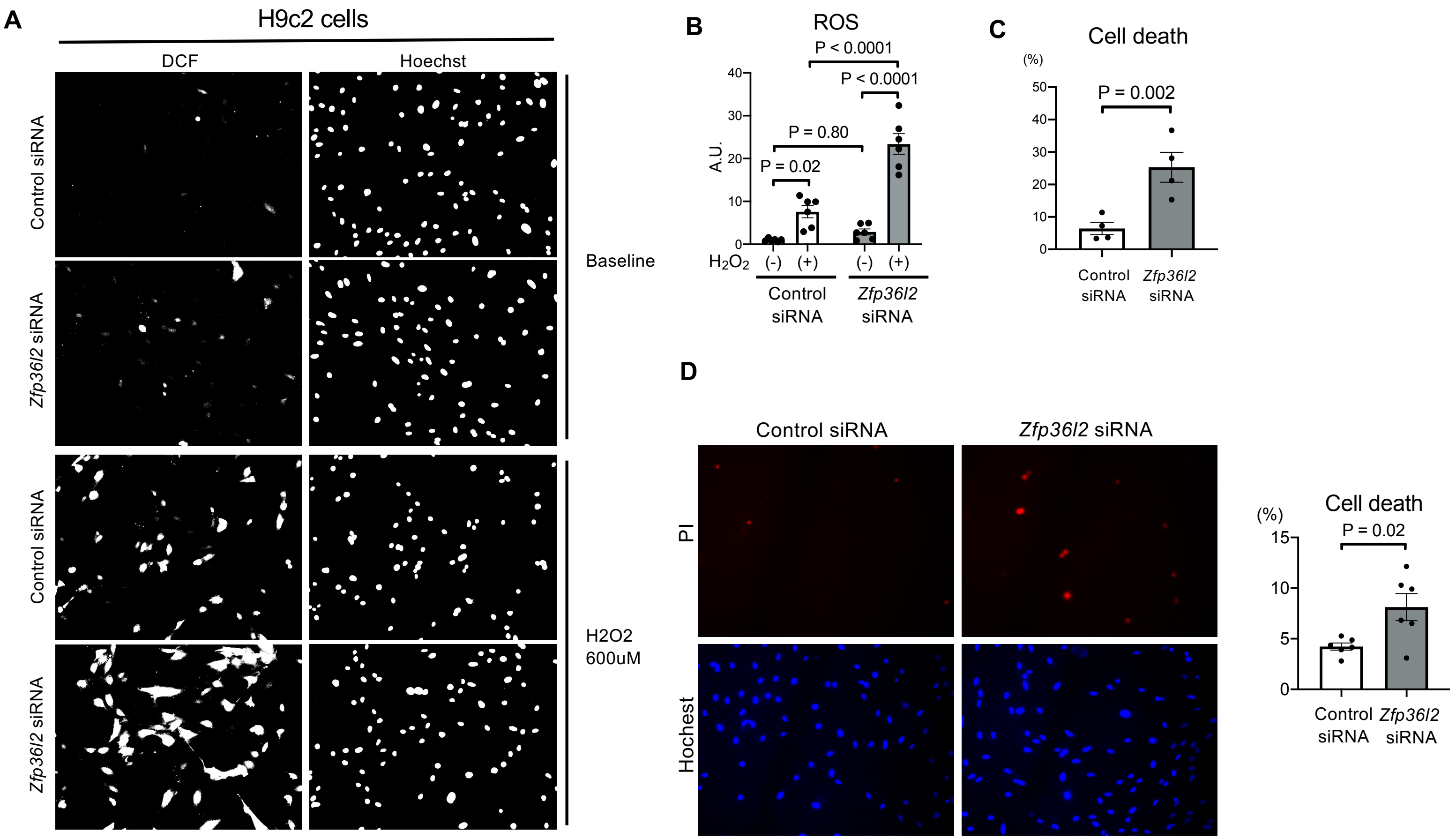

Supplemental Figure 11. KD of *Zfp36l2* causes an exaggerated ROS production and cell death.
(A,B) ROS levels as assessed by DCF staining in H9c2 cells after treatment with H_2O_2 and control or *Zfp36l2* siRNA (*n* = 6). **(C)** Cell death in H9c2 cells after treatment with H_2O_2 and control or *Zfp36l2* siRNA (*n* = 4). **(D)** Cell death in H9c2 cells after treatment with etoposide and control or *Zfp36l2* siRNA (*n* = 6). Data were analyzed by 2-way ANOVA with Tukey's test for multiple group comparison (B) or unpaired Student *t* test (C,D).

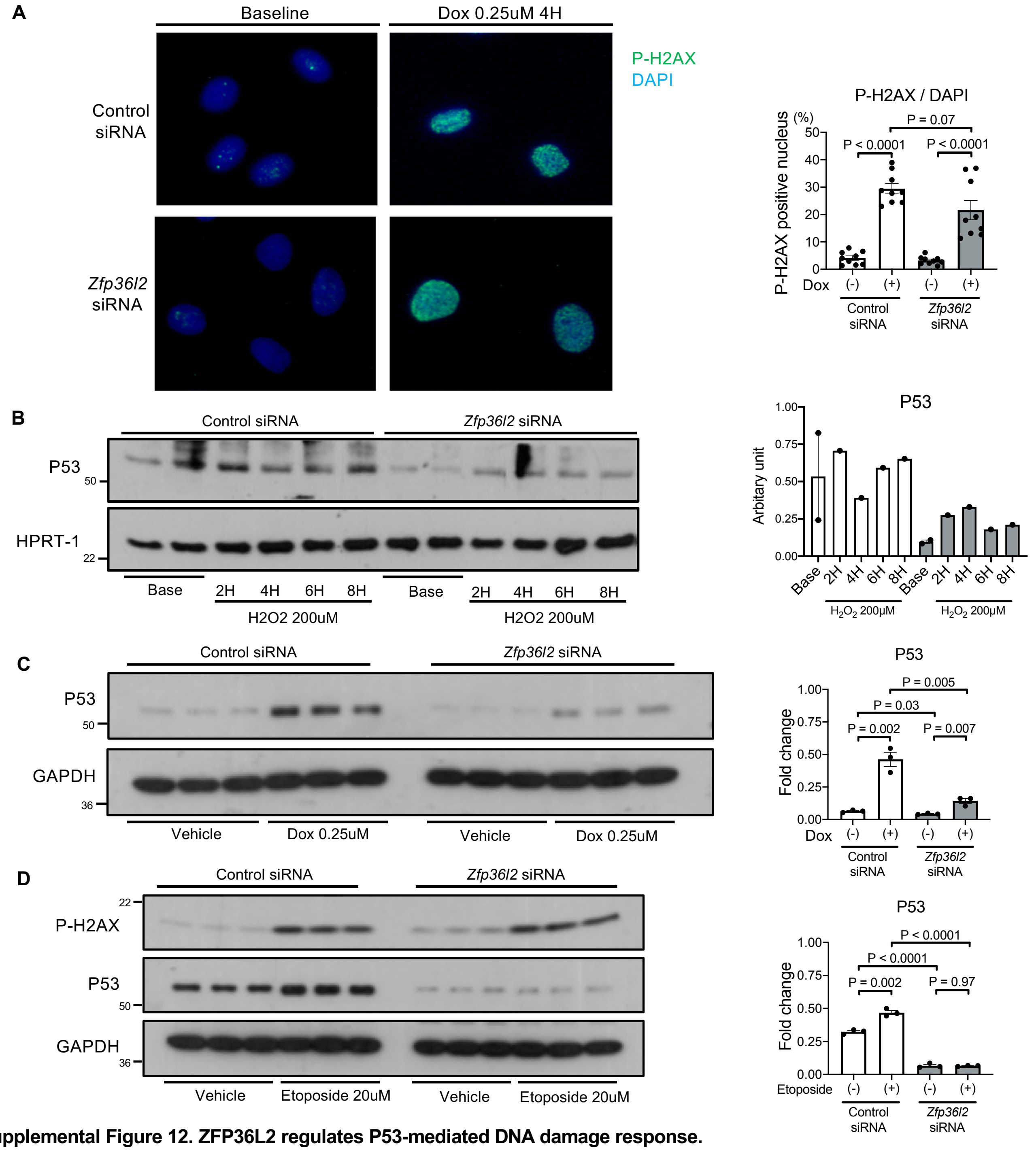

Supplemental Figure 12. ZFP36L2 regulates P53-mediated DNA damage response.
(A) Levels of phosphorylated histone H2AX, a marker of DNA strand break, in control and *Zfp36l2* siRNA treated H9c2 cells after 0.25 μM doxorubicin (Dox) for 4 hours (*n* = 9). P53 protein levels in H9c2 cells with *Zfp36l2* KD after treatment with H_2O_2 (**B**), or Dox (**C**) for 16 hours (*n* = 1-2 for **B** and *n* = 3 for **C**). **(D)** Levels of phosphorylated histone H2AX and P53 protein in H9c2 cells with *Zfp36l2* KD treated with etoposide for 16 hours (*n* = 3). Data were analyzed by 2-way ANOVA with Tukey's test for multiple group comparison.

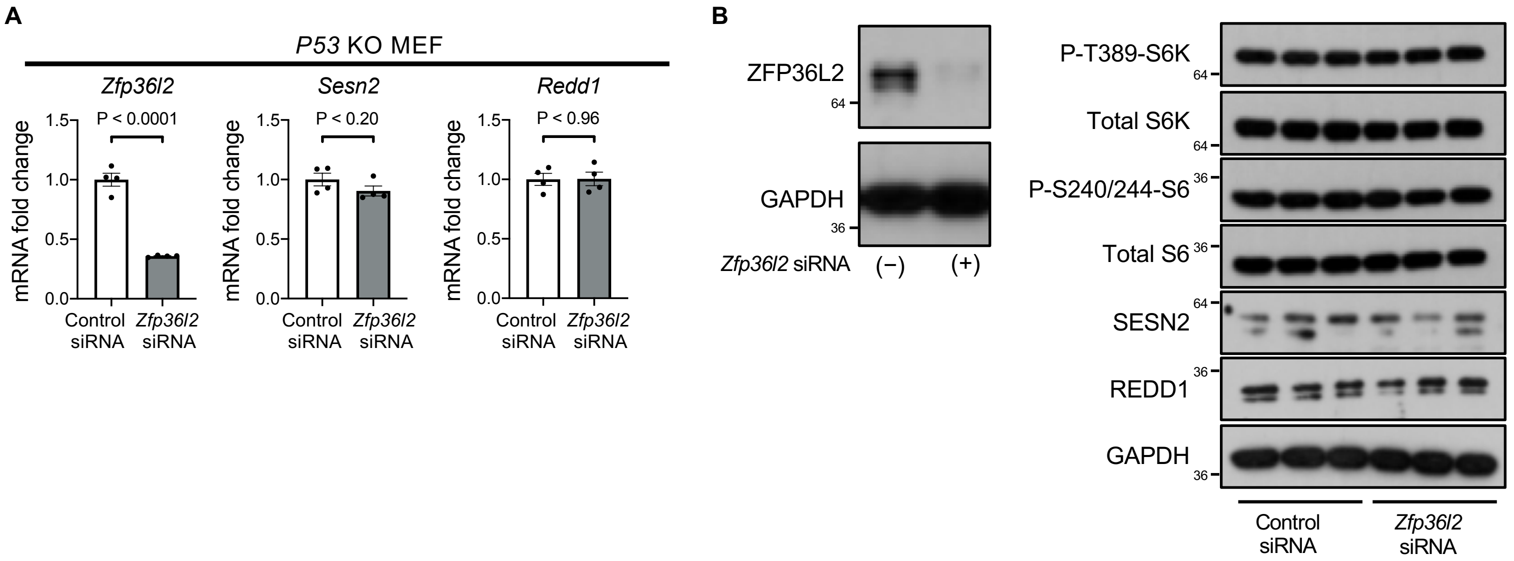

Supplemental Figure 13. Analysis of SESN2 and REDD1 mRNA, protein levels and mTOR activity in *P53* KO MEFs treated with control or *Zfp36l2* siRNA. **(A)** mRNA levels of *Zfp36l2*, *Sesn2* and *Redd1* in *P53* KO MEFs treated with *Zfp36l2* siRNA, illustrating lack of a response in *Sesn2* and *Redd1* with *Zfp36l2* KD in these cells (n = 4). **(B)** T389 phosphorylation of P70S6K, S240/244 phosphorylation of S6, and SESN2 and REDD1 protein levels in *p53* KO MEFs treated with control or *Zfp36l2* siRNA (n = 3). Data were analyzed by unpaired Student t test.

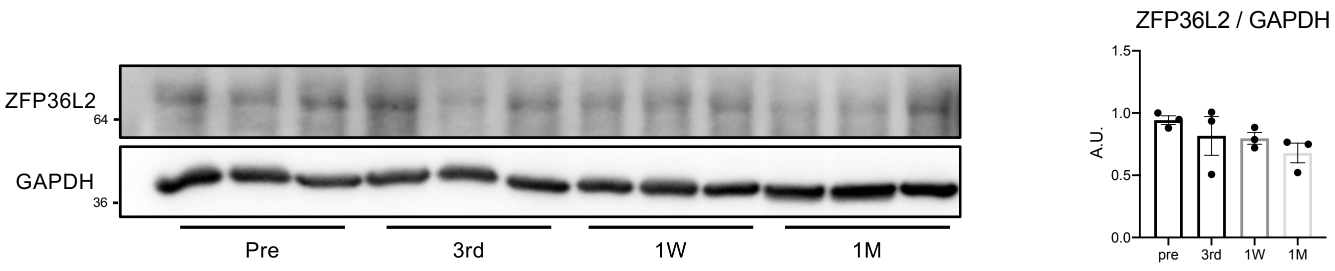

Supplemental Figure 14. ZFP36L2 protein levels in the heart before pregnancy (Pre), during the 3rd trimester (3rd), and one week (1W) and one month (1M) after delivery. Summary of the data is shown in the graph next to the image. *n* = 3 in each group. P = 0.32 based on one-way ANOVA.

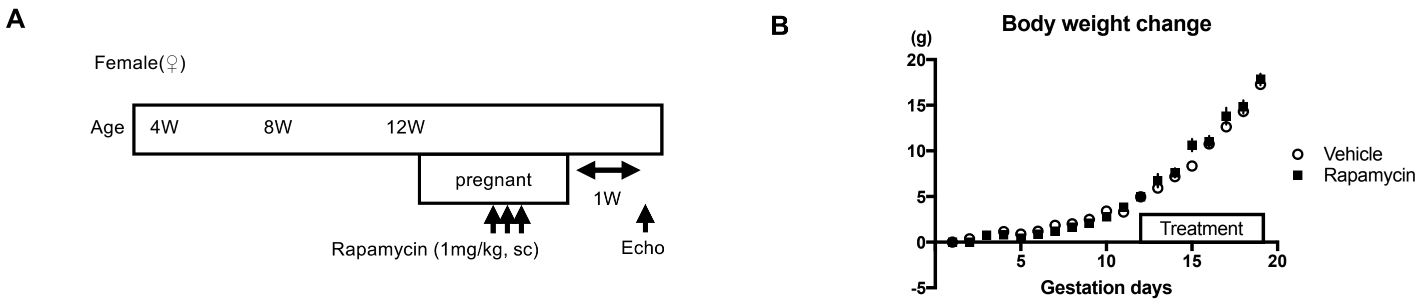

Supplemental Figure 15. Protocol and mouse body weight after rapamycin treatment.
(A) Protocol for treatment of mice with rapamycin before and during pregnancy. **(B)** Body weight of pregnant mice treated with control or rapamycin, indicating no change in body weight with rapamycin treatment.

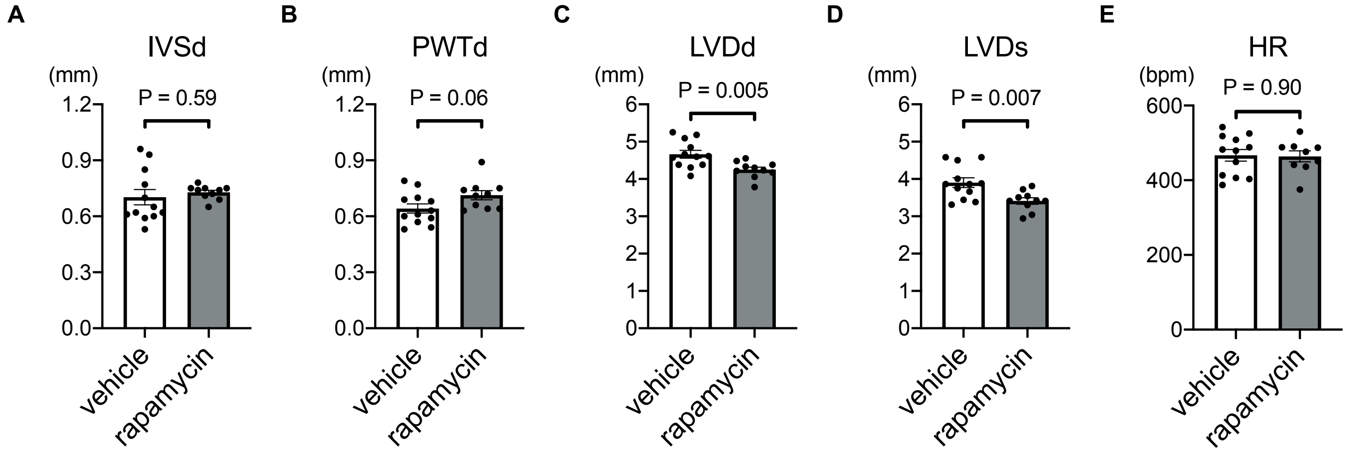

Supplemental Figure 16. Echo parameters and heart rate in *Zfp36l2* KO mice treated with vehicle or rapamycin (*n* = 10-12).
Interventricular septal wall thickness end diastole (IVSd, **A**), posterior wall thickness end diastole (PWTd, **B**), left ventriculat diameter end diastole (LVDd, **C**), left ventricular diameter end systole (LVDs, **D**) and heart rate (HR, **E**) in *Zfp36l2* KO mice treated with vehicle or rapamycin. Data were analyzed by unpaired Student *t* test.

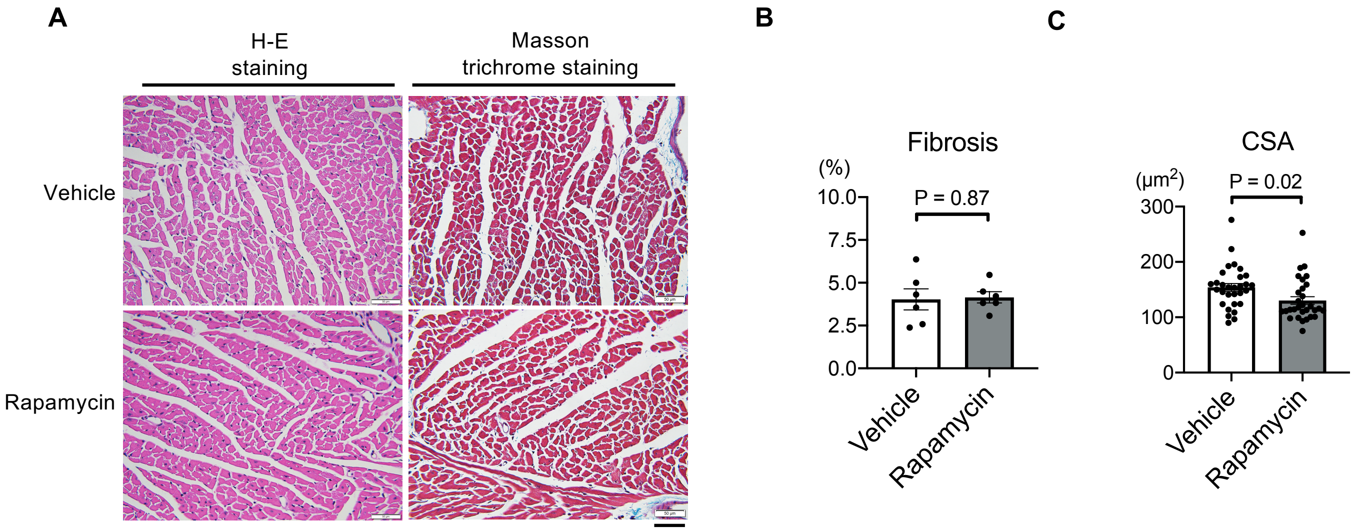

Supplemental Figure 17. Degree of fibrosis and cross sectional area in the hearts of *Zfp36l2* KO mice after treatment with rapamycin. Histological images **(A)**, degree of fibrosis **(B)** and cross sectional area **(C)** of hearts of mice with *Zfp36l2* deletion after treatment with rapamycin (n = 3 mice, and 10 individual cardiomyocytes for CSA and 2 areas for fibrosis graph). The scale indicates 50 μm. Data were analyzed by unpaired Student t test.

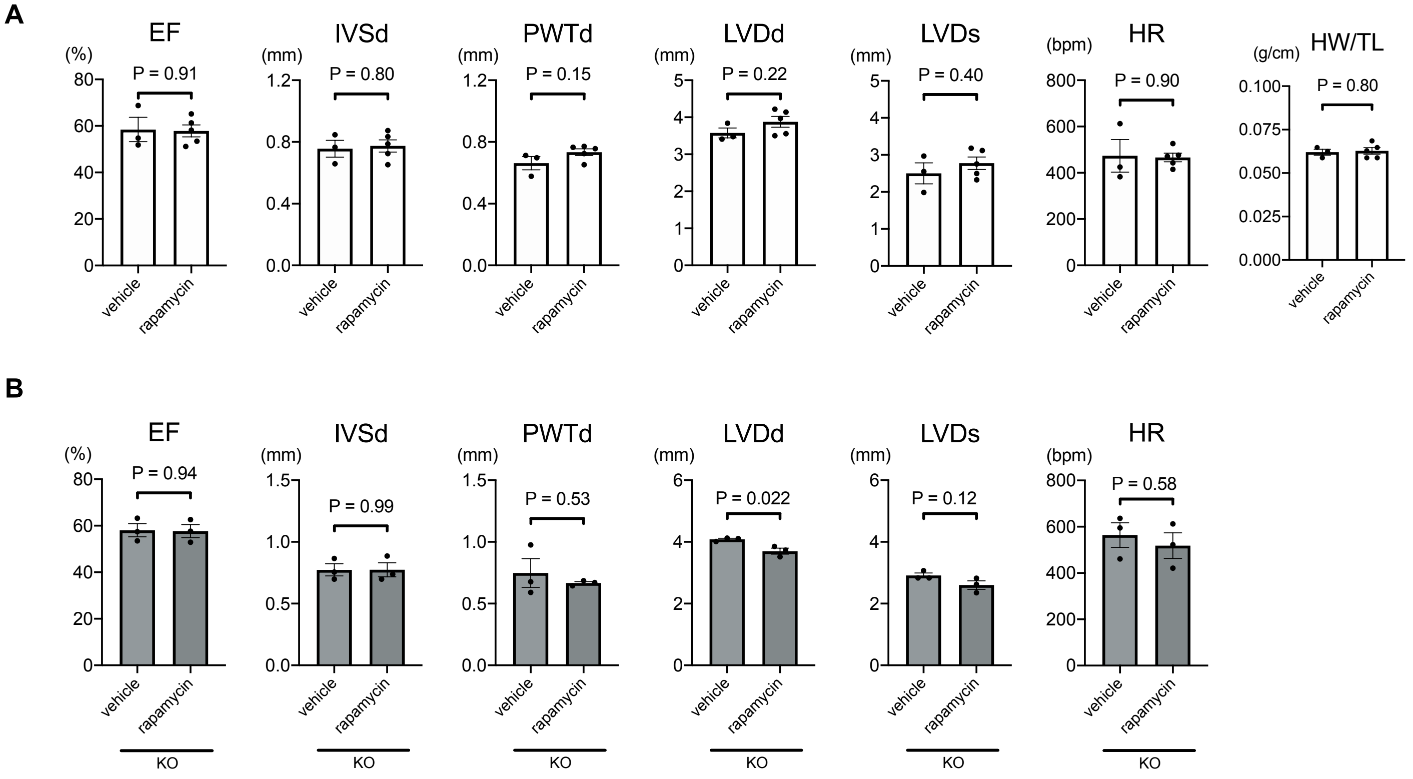

Supplemental Figure 18. Echo parameters pregnant WT mice (A, *n* = 3-5) and in *Zfp36l2* KO mice without pregnancy (B, *n* = 3) after treatment with rapamycin or vehicle. Data were analyzed by unpaired Student *t* test.

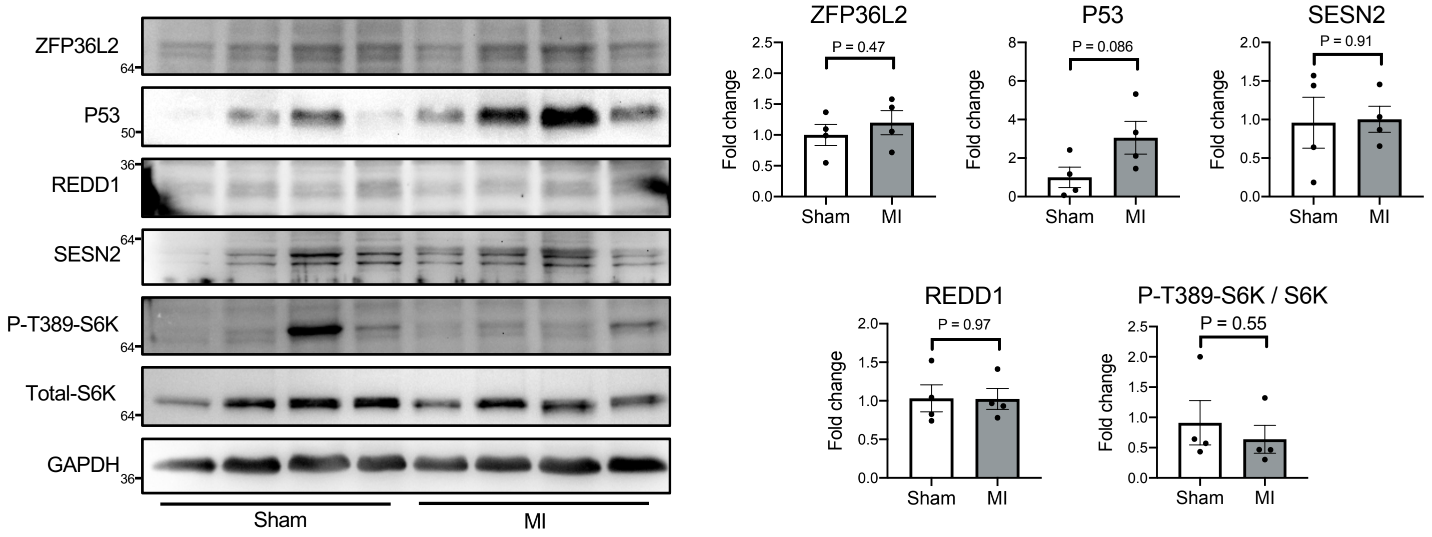

Supplemental Figure 19. Protein levels of the components of the ZFP36L2-P53-REDD1/SESN2-mTOR pathway in the hearts of mice after sham operation or MI. Summary graphs are provided on the right side (*n* = 4). Data were analyzed by unpaired Student *t* test.

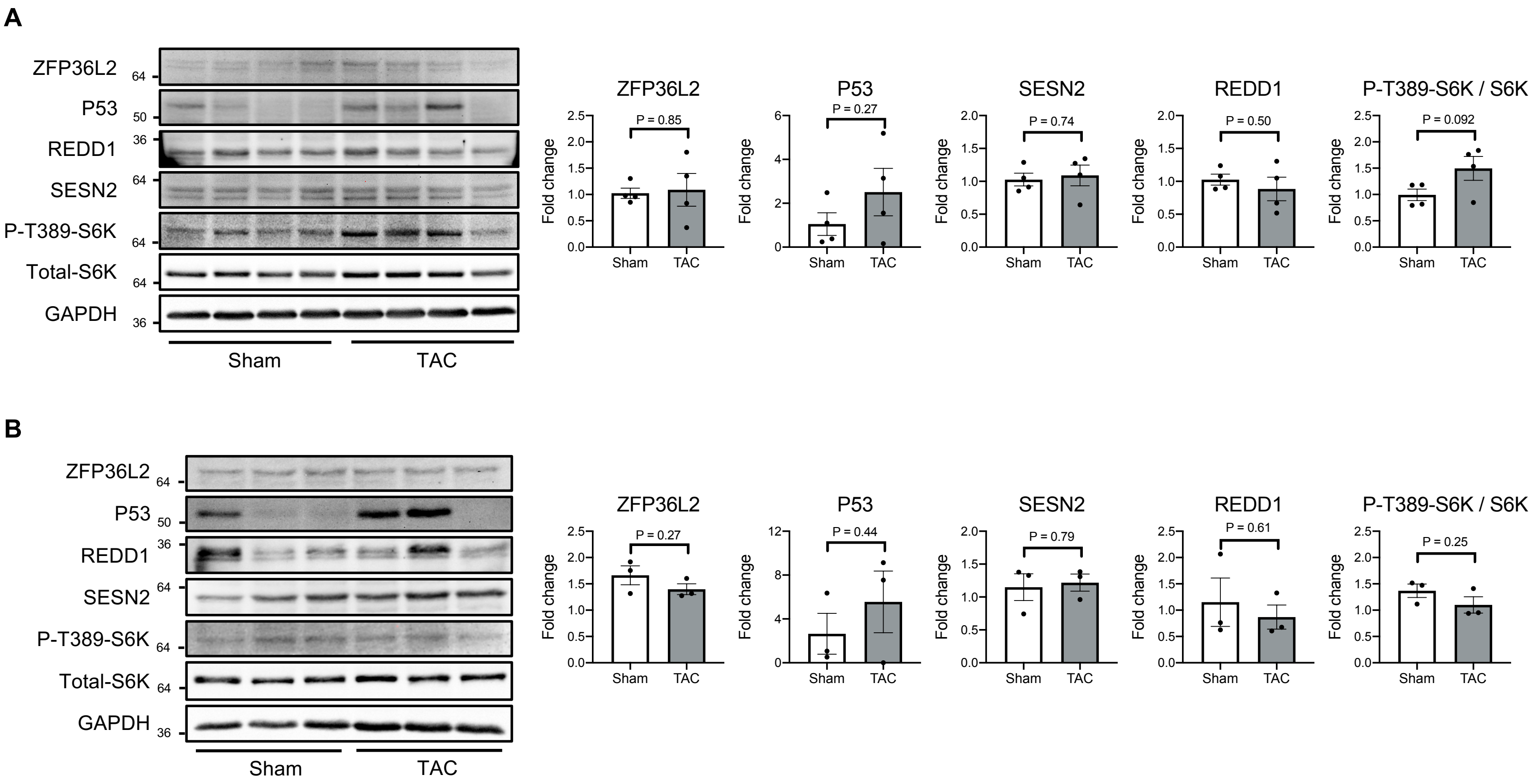

Supplemental Figure 20. Protein levels of the components of the ZFP36L2-P53-REDD1/SESN2-mTOR pathway in the hearts of female (A, *n* = 4) and male (B, *n* = 3) mice after sham operation or TAC. Summary graphs are provided on the right side. Data were analyzed by unpaired Student *t* test.

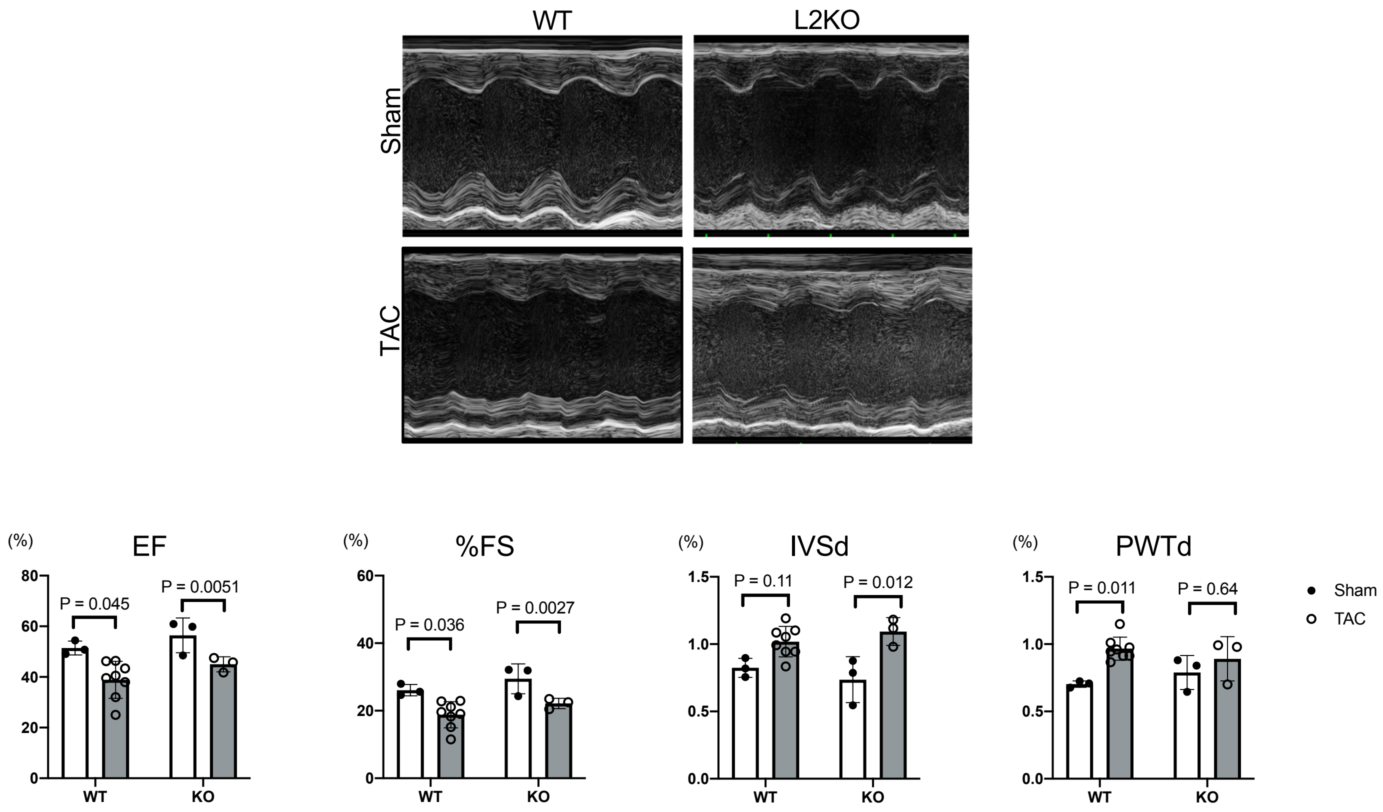

Two-way ANOVA
Interaction: P = 0.87
Genotype factor: P = 0.11
Surgery facor: P = 0.003

Two-way ANOVA
Interaction: P = 0.99
Genotype factor: P = 0.90
Surgery facor: P = 0.0016

Two-way ANOVA
Interaction: P = 0.21
Genotype factor: P = 0.92
Surgery facor: P = 0.0007

Two-way ANOVA
Interaction: P = 0.16
Genotype factor: P = 0.93
Surgery facor: P = 0.0054

Supplemental Figure 21. Cardiac function in *Zfp36l2* KO mice and their littermate controls 4 weeks after TAC. N = 3-8 in each group.
